# Supplementary material for: Connective tissue fibroblasts from highly regenerative mammals are refractory to ROS-induced cellular senescence
Source: Nat Commun. 2019 Sep 27;10:4400. doi: 10.1038/s41467-019-12398-w (PMC6764955; doi:10.1038/s41467-019-12398-w)
Supplement: Supplementary file 1 — Supplementary information_new [file 41467_2019_12398_MOESM1_ESM.pdf]

Supplementary Information

Connective tissue fibroblasts from highly regenerative mammals are refractory to ROS-induced cellular senescence

Sandeep Saxena[1], Hemendra Vekaria[2,3], Patrick G. Sullivan[2,3,4] and Ashley W. Seifert[1,3†]

[1]*Department of Biology, University of Kentucky, Lexington, KY 40506, USA*
[2]*Department of Neuroscience, University of Kentucky, Lexington, KY 40506, USA*
[3]*The Spinal Cord and Brain Injury Research Center (SCoBIRC), University of Kentucky, Lexington, KY 40506, USA*
[4]*Lexington VA Medical Center, Lexington, KY 40506, USA*

Supplementary Information

Supplementary Figures

Supplementary Figure 1.

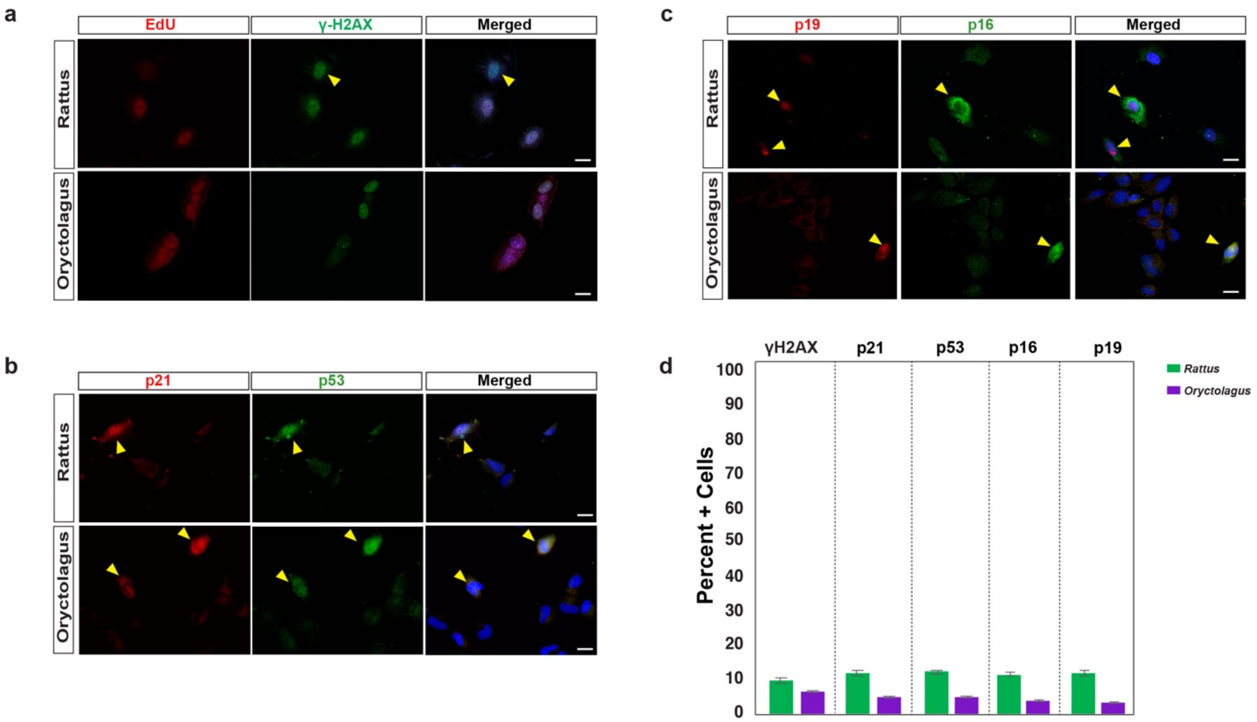

Rattus **and** ***Oryctolagus*** **have few percent+ cells for senescent markers at P2** (a-c) Representative images for *Rattus* and *Oryctolagus* fibroblasts at P2 (*n*=4/species) double labeled with γ-H2AX and EdU (a), p21 and p53 (b) and p16 and p19 (c). Yellow arrows indicate the positive senescent cells. (d) Percent positive cells were counted for γ-H2AX, p21, p53, p16 and p19. Scale bars in (a-c) = 20µm. Error bars = S.E.M. Source data are provided as a Source Data file.

Supplementary Information

Supplementary Figure 2.

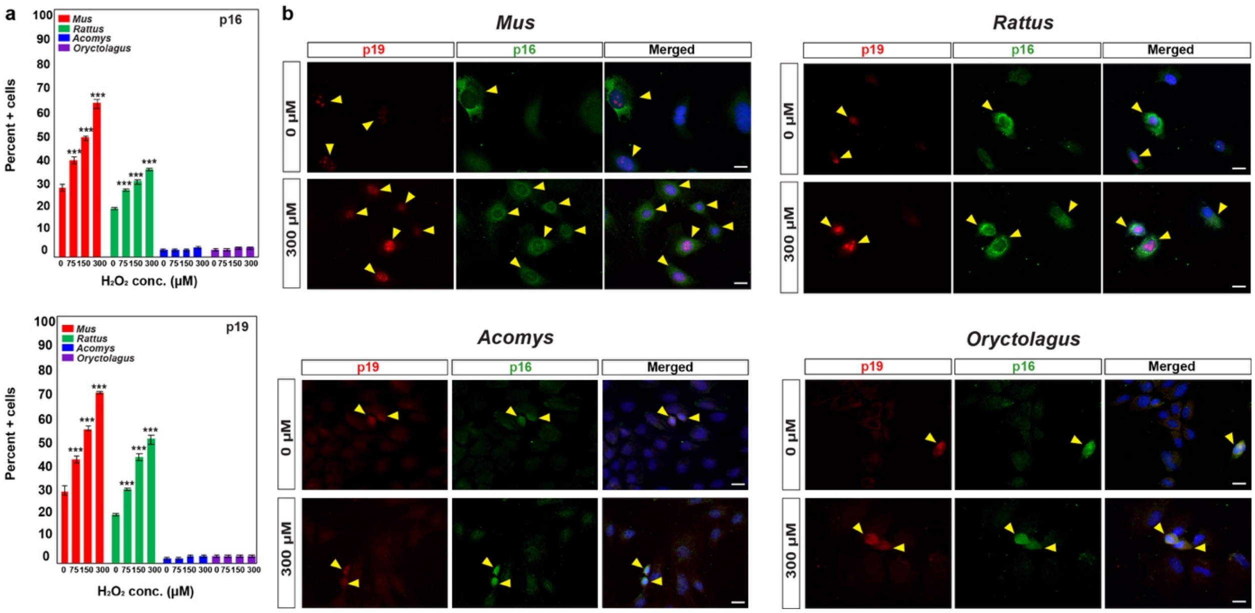

Fibroblasts from regenerating animals do not significantly increase p16 and p19 upon H_2O_2 exposure. (a-b) Fibroblasts (*n*=4/species) from all the four species were treated with different sub-lethal dose of H_2O_2 (0µM-control, 75µM, 150µM and 300µM H_2O_2) for 2hrs and cultured for 48hrs. (a) The percentage of p16+ and p19+ cells were significantly increased in *Rattus* and *Mus* while these markers did not significantly change in *Acomys* and *Oryctolagus* (p16+ cells, ANOVA, F=757.2077, P<.0001 and p19+ cells, ANOVA, F=1064.6167, P<.0001). Error bars shows S.E.M. (b) Representative images showing positive staining for p16 and p19 (yellow arrows). DAPI = blue in merged panels. ***P<0.0001, ** P<0.001 and * P<0.05. Scale bars = 20µm. Error bars = S.E.M. Source data are provided as a Source Data file.

Supplementary Information

Supplementary Figure 3.

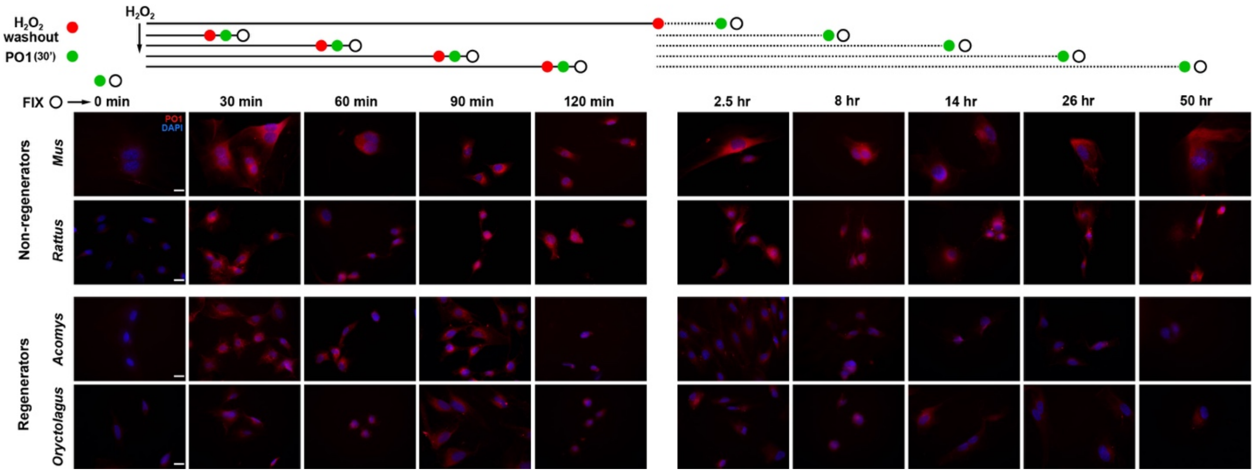

Rapid detoxification of H_2O_2 in fibroblasts from regenerators compared to non-regenerators. PO1 fluorescence indicating intracellular H_2O_2 level in fibroblasts from regenerating (*Acomys* and *Oryctolagus*) and non-regenerating mammals (*Mus* and *Rattus*) treated with PBS (control) or 300µM H_2O_2 for different time periods (*n*=4/species). Schematic at top indicates time post exposure and H_2O_2 washout (red circle), followed by 30mins PO1 treatment (green circle) and fixation (open circle). Time points post exposure include time until fixation. PO1 fluorescence in untreated cells indicated physiological levels of H_2O_2 across all cells which were not significantly different among species. In response to exogenous H_2O_2 intracellular H_2O_2 remained high and elevated in fibroblasts from non-regenerating species even at 48hrs post H_2O_2 washout. Intracellular H_2O_2 increased in fibroblasts from regenerating species but was reduced near baseline levels after H_2O_2 washout. Scale bars are representative for all panels and = 20µm.

Supplementary Information

Supplementary Figure 4.

(a) GPx1

```
Rattus         1 MSAARLSAVAQSTVYAFSARPLAGGEPVSLGSLRGKVLLIENVASLUGTTTRDYTEMNDL
Acomys         1 --------------------------------------------------------MNDL
Mus            1 MCAARLSAAAQSTVYAFSARPLTGGEPVSLGSLRGKVLLIENVASLUGTTIRDYTEMNDL
Oryctolagus    1 -MCAARMAAAAQSVYSFSAHPLAGGEPVNLGSLRGKVLLIENVASLUGTTVRDYTQMNEL

Rattus        61 QKRLGPRGLVVLGFPCNQFGHQENGKNEEILNSLKYVRPGGGFEPNFTLFEKCEVNGEKA
Acomys         5 QKRLGPRGLRVLGFPCNQFGHQENAKNEEILNSLKYIRPGGGFEPNFTLFEKCEVNGEKA
Mus           61 QKRLGPRGLVVLGFPCNQFGHQENGKNEEILNSLKYVRPGGGFEPNFTLFEKCEVNGEKA
Oryctolagus   60 QERLGPRALVVLGFPCNQFGHQENAKNEEILNSLKYVRPGGGFEPNFMLFQKCEVNGAKA

Rattus       121 HPLFTFLRNALPAPSDDPTALMTDPKYIIWSPVCRNDISWNFEKFLVGPDGVPVRRYSRR
Acomys        65 HPLFRFLREALPAPSDEPTALMTDPKYIIWSPVCRNDVAWNFEKFLVGPDGVPVRRYSRR
Mus          121 HPLFTFLRNALPTPSDDPTALMTDPKYIIWSPVCRNDIAWNFEKFLVGPDGVPVRRYSRR
Oryctolagus  120 SPLFAFLREALPPPSDDPTALMTDPKFITWCPVCRNDVSWSFEKFLVGPDGVPVRRYSRR

Rattus       181 FRTIDIEPDIEALLSKQPSNP
Acomys       125 FRTIDIEPDIEALLSQQPSSP
Mus          181 FRTIDIEPDIETLLSQQSGNS
Oryctolagus  180 FPTIDIEPDIQALLSKGSGGA
```

(b) GPx2

```
Rattus         1 MAYIAKSFYDLSAIGLDGEKIDFNTFRGRAVLIENVASLUGTTTRDYTQLNELQCRFPRR
Acomys         1 ---------------------------------------------------MVAAACPVR
Mus            1 MAYIAKSFYDLSAVGLDGEKIDFNTFRGRAVLIENVASLUGTTTRDYNQLNELQCRFPRR
Oryctolagus    1 MAYIAKSFYDLTAVSLDGEKVDFNTFRGRAVLIENVASLUGTTTRDFTQLNELQCRYPRR

Rattus        61 LVVLGFPCNQFGHQENCQNEEILNSLKYVRPGGGFQPTFSLTQKCDVNGQNQHPVFAYLK
Acomys        10 LVILGFPCNQFGHQENCQNEEILNSLKYVRPGRGYQPTFTLTQKCEVNGQNEHPVFAYLK
Mus           61 LVVLGFPCNQFGHQENCQNEEILNSLKYVRPGGGYQPTFSLTQKCDVNGQNEHPVFAYLK
Oryctolagus   61 LVVLGFPCNQFGHQENCQDEEILNSLKYVRPGGGYQPTFTLVQKCEVNGQNQHPVFTYLK

Rattus       121 DKLPYPYDDPFSLMTDPKLIIWSPVRRSDVSWNFEKFLIGPEGEPFRRYSRTFQTINIEP
Acomys        70 DKLPYPYDDPFSLMTDPKLIMWSPVRRSDVAWNFEKFLIGPEGEPFRRYSRTFQTINIEP
Mus          121 DKLPYPYDDPFSLMTDPKLIIWSPVRRSDVSWNFEKFLIGPEGEPFRRYSRSFQTINIEP
Oryctolagus  121 DKLPYPHDDPFSLMTDPKFIIWSPVRRSDVAWNFEKFLIGPEGEPFRRYSRTFPTINIEP

Rattus       181 DIKRLLKVAI
Acomys       130 DIKRLLKVAI
Mus          181 DIKRLLKVAI
Oryctolagus  181 DIKRLLKVAI
```

Supplementary Information

(c) GPx3

```
Rattus          1  MARILLRASCLLSLLLAGFVPPGRGQEKSKTDCHGGMSGTIYEYGALTIDGEEYIPFKQYA
Acomys          1  MARLLRASCLLSLLLAGFIPPGRGQEKSKTDCNG--------------------------
Mus             1  MARILLRASCLLSLLLAGFVPPGRGQEKSKTDCHGGMSGTIYEYGALTIDGEEYIPFKQYA
Oryctolagus     1  MARLLRASCLLSLLLAGFVPPGRGQEKSKTDCHGGVSGTIYEYGALTIDGEEYIPFKQYA

Rattus         61  GKYILFVNVASYUGLTDQYLELNALQEELGPFGLVILGFPCNQFGKQEPGENSEILPSLK
Acomys         35  -------------GVSDQYLELNALQEELGPFGLVILGFPCNQFGKQEPGENSEILPSLK
Mus            61  GKYILFVNVASYUGLTDQYLELNALQEELGPFGLVILGFPSNQFGKQEPGENSEILPSLK
Oryctolagus    61  GKYVLFVNVATYUGLTGQYVELNALQEELAPFGLVILGFPCNQFGKQEPGENSEILPALK

Rattus        121  YVRPGGGFVPNFQLFEKGDVNGEKEQKFYTFLKNSCPPTAELLGSPGRLFWEPMKIHDIR
Acomys         82  YVRPGGGFVPNFQLFEKGDVNGVKEQKFYTFLKNSCPPTAELLGSPGRLFWEPMKIHDIR
Mus           121  YVRPGGGFVPNFQLFEKGDVNGEKEQKFYTFLKNSCPPTAELLGSPGRLFWEPMKIHDIR
Oryctolagus   121  YVRPGGGFVPNFQLFEKGDVNGDKEQKVYTFLKNSCPPTSELLGSPNRLFWEPMKMHDVR

Rattus        181  WNFEKFLVGPDGIPIMRWYHRTTVSNVKMDILSYMRRQAALGARGK
Acomys        142  WNFEKFLVGPDGIPVMRWYHRTTVSNVKMDILSYMRRQAALGARGK
Mus           181  WNFEKFLVGPDGIPVMRWYHRTTVSNVKMDILSYMRRQAALSARGK
Oryctolagus   181  WNFEKFLVGPDGVPIMRWYHRATVSNVKMDILAYMRRQAAMGAKGK
```

Supplementary Information

(d) Catalase

```
Rattus          1  MADSRDPASDQMKQWKEQRAPQKPDVLTTGGGNPIGDKLNIMTAGPRGPLLVQDVVFTDE
Acomys          1  MADSRDPASDQMKQWKEQRALQKPDVLTTGGGNPIGDKLNVMTAGSRGPLLVQDVVFTDE
Mus             1  MSDSRDPASDQMKQWKEQRASQRPDVLTTGGGNPIGDKLNIMTAGSRGPLLVQDVVFTDE
Oryctolagus     1  MGDSRDPASDQMKKWKEQRGAQKPDVLTTGAGNPIGDKLNIITAGPRGPLLVQDVVFTDE

Rattus         61  MAHFDRERIPERVVHAKGAGAFGYFEVTHDITRYSKAKVFEHIGKRTPIAVRFSTVAGES
Acomys         61  MAHFDRERIPERVVHAKGAGAFGYFEVTHDITSYCKAKVFEHIGKRTPIAVRFSTVAGES
Mus            61  MAHFDRERIPERVVHAKGAGAFGYFEVTHDITRYSKAKVFEHIGKRTPIAVRFSTVTGES
Oryctolagus    61  MAHFDRERIPERVVHAKGTGAFGYFEVTHDITRYSKAKVFEHIGKKTPIAVRFSTVAGES

Rattus        121  GSADTVRDPRGFAVKFYTEDGNWDLVGNNTPIFFIRDAMLFPSFIHSQKRNPQTHLKDPD
Acomys        121  GSADTVRDPRGFAVKFYTEDGNWDLVGNNTPIFFIRDAILFPSFIHSQKRNPQTHLKDPD
Mus           121  GSADTVRDPRGFAVKFYTEDGNWDLVGNNTPIFFIRDAILFPSFIHSQKRNPQTHLKDPD
Oryctolagus   121  GSADTVRDPRGFAVKFYTEDGNWDLVGNNTPIFFIRDALLFPSFIHSQKRNPQTHLKDPD

Rattus        181  MVWDFWSLCPESLHQVTFLFSDRGIPDGHRHMNGYGSHTFKLVNANGEAVYCKFHYKTDQ
Acomys        181  MVWDFWSLRPESLHQVSFLFSDRGIPDGHRHMNGYGSHTFKLVNANGEAVYCKFHYKTDQ
Mus           181  MVWDFWSLRPESLHQVSFLFSDRGIPDGHRHMNGYGSHTFKLVNADGEAVYCKFHYKTDQ
Oryctolagus   181  MVWDFWSLRPESLHQVSFLFSDRGIPDGHRHMNGYGSHTFKLVNASGEAVYCKFHYKTDQ

Rattus        241  GIKNLPVEEAGRLAQEDPDYGLRDLFNAIASGNYPSWTFYIQVMTFKEAETFPFNPFDLT
Acomys        241  GIKNLPVGEAGRLAQEDPDYGLRDLFNAIANGNYPTWTFYIQVMTFKEAETFPFNPFDLT
Mus           241  GIKNLPVGEAGRLAQEDPDYGLRDLFNAIANGNYPSWTFYIQVMTFKEAETFPFNPFDLT
Oryctolagus   241  GIKNLPVADAARISQEDPDYGIRDLFNAIATGNYPSWTFYIQVMTFDQAETFPFNPFDLT

Rattus        301  KVWPHKDYPLIPVGKLVLNRNPANYFAEVEQMAFDPSNMPPGIEPSPDKMLQGRLFAYPD
Acomys        301  KVWPHKDYPLIPVGKLVLNRNPVNYFAEVEQMAFDPSNMPPGIEPSPDKMLQGRLFAYPD
Mus           301  KVWPHKDYPLIPVGKLVLNKNPVNYFAEVEQMAFDPSNMPPGIEPSPDKMLQGRLFAYPD
Oryctolagus   301  KIWPHKDYPLIPVGKLVLNRNPVNYFAEIEQLAFDPSNMPPGIEPSPDKMLQGRLFSYPD

Rattus        361  THRHRLGPNYLQIPVNCPYRARVANYQRDGPMCMHDNQGGAPNYYPNSFSAPEQQGSALE
Acomys        361  THRHRLGPNYLQIPVNCPYRARVANYQRDGPMCMHDNQGGAPNYYPNSFSAPEQQRSALE
Mus           361  THRHRLGPNYLQIPVNCPYRARVANYQRDGPMCMHDNQGGAPNYYPNSFSAPEQQRSALE
Oryctolagus   361  THRHRLGPNYLQIPVNCPYRARVANYQRDGPMCITDNQGGAPNYYPNSFSAPDQQPSALE

Rattus        421  HHSQCSADVKRFNSANEDNVTQVRTFYTKVLNEEERKRLCENIANHLKDAQLFIQRKAVK
Acomys        421  HGAQCSVDVKRFNSANEDNVTQVRTFYTKVLNEEERKRLCENIAGHLKDAQLFIQKKAVK
Mus           421  HSVQCAVDVKRFNSANEDNVTQVRTFYTKVLNEEERKRLCENIAGHLKDAQLFIQKKAVK
Oryctolagus   421  HGTRCSGDVQRFNSTNEDNVSQVRDFYVKVLNEEERKRLCENIAGHLKDAQLFIQKKAVK

Rattus        481  NFTDVHPDYGARVQALLDQYNSQKPKNAIHTYVQAGSHIAAKGKANL
Acomys        481  NFSDVHPDYGARIQALLDKYNAEKPKNAIHTYMQAGSHLAAKEKANL
Mus           481  NFTDVHPDYGARIQALLDKYNAEKPKNAIHTYTQAGSHMAAKGKANL
Oryctolagus   481  NFSDVHPDYGARIQALLDKYNAEKPKNAIHTFVQSGSHLTAKEKANL
```

Supplementary Information

(e)

**Percent identity matrix score to *Mus* across all species**			
**Protein name**	***Acomys***	***Rattus***	***Oryctolagus***
GPx1	91.03	94.52	82.50
GPx2	89.92	97.36	91.05
GPx3	94.65	98.67	90.26
Catalase	96.77	95.06	90.89

High sequence conservation of GPx (1-3) and catalase protein sequences across all four species examined in this study. The fasta sequences of GPx1 (a), GPx2 (b), GPx3 (c) and catalase (d) were aligned using the multiple sequence alignment tool clustal omega. (a-c) The blue color marked amino acid 'U' is important for GPx activity. The 5' missing sequence for *Acomys* is an assembly artifact. (e) The percent identity matrix score for GPx1 compared to *Mus* was 91.03 for *Acomys*, 94.52 for *Rattus* and 82.50 for *Oryctolagus,* while it was ≥ 90 for GPx2, GPx3 and catalase.

Supplementary Information

Supplementary Figure 5.

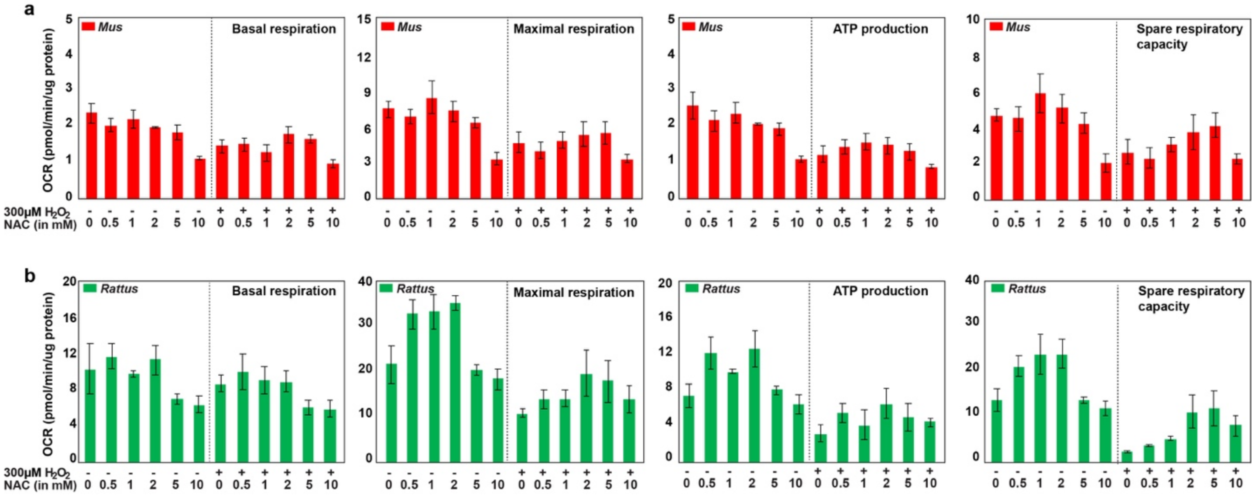

Optimized NAC pre-treatment protects *Mus* and *Rattus* cells from stress-induced senescence. (a-b) The 1hr pre-treatment with different concentrations of NAC (0mM-control, 0.5mM, 1mM 2mM, 5mM and 10mM NAC) showed varying levels of protection in *Mus* (a) and *Rattus* (b) cells in response to exogenous H_2O_2 treatment (*n*=3/species). Indicated parameters were measured via mitochondrial stress testing. The normalized OCR were analyzed for non-treated and NAC+ H_2O_2 (pre-treatment with different concentrations of NAC followed by 2hrs of 300µM H_2O_2 treatment) treated samples in *Mus* (a) and *Rattus* (b) for all the measured parameters such as basal respiration, maximal respiration, ATP production, and spare respiratory capacity. The data support 2mM NAC as the optimal dose across *Mus* and *Rattus*. Source data are provided as a Source Data file.

Supplementary Information

Supplementary Figure 6.

(a) **CDKN2A (p16)**

```
Rattus         1 ---------MESSADRLARAAALGREHEVRALLEAGASPNAPNTFGRTPIQVMMMGNVKVA
Acomys         1 MESLKDQQPDSLGDQLSRAAAQGRVHEVRTLLEAGVSPNAPNSFGRTPIQVMMMGNTQIA
Mus            1 ---------MESAADRLARAAAQGRVHDVRALLEAGVSPNAPNSFGRTPIQVMMMGNVHVA
Oryctolagus    1 ---------MEPSADRLATAAARGRVEEVRALLEAGVPPDAPNRYGRSAIQVMMMGSARVA

Rattus        53 ALLLSYGADSNCEDPTTLSRPVHDAAREGFLDTLVVLHQAGARLDVRDAWGRLPLDLALE
Acomys        61 NLLLFYGADPNCEDPVTLSRPVHDAAREGFLDTLVALHQAGARLDVRDAFDRLPMDLAQE
Mus           53 ALLLNYGADSNCEDPTTFSRPVHDAAREGFLDTLVVLHGSGARLDVRDAWGRLPLDLAQE
Oryctolagus   53 ELLLLHGAEPNCADPATLSRPVHDAAREGFLDTLVALHRAGARLDVRDAGGRLPVDLAEE

Rattus       113 RGHHDVVRYLRYLLSSAGNVSRVTDRHNFCSSTPRCLGLRGQPPKQR---------
Acomys       121 QGHRDVLLYLQVAGGATAQASPTTGTASAYPPPDPSDLPDLEKQHIK---------
Mus          113 RGHQDIVRYLRSAGCSLCSAGWSLCTAGNVAQTDGHSFSSSTPRALELRGQSQEQS
Oryctolagus  113 RGHRDVARYLRAAEGGNHARARAVEGTADTPNSNDL--------------------
```

(b) **CDKN2D (p19)**

```
Rattus         1 MLLEEVSVGDRLSGAAARGDVQEVRRLLHRELVHPDALNRFGKTALQVMMFGSPAVALEL
Acomys         1 MLLEEVCVGDRLSGAAARGDVQEVRRLLHRELVHPDALNRFGKTALQVMMFGSPAVALEL
Mus            1 MLLEEVCVGDRLSGAAARGDVQEVRRLLHRELVHPDALNRFGKTALQVMMFGSPAVALEL
Oryctolagus    1 MLLEEVRAGDRLSGAAARGDVQEVRRLLHRELVHPDALNRFGKTALQVMMFGSPSIALEL

Rattus        61 LKQGASPNVQDASGTSPVHDAARTGFLDTLKVLVEHGADVNTLDSTGSLPIHLAIREGHS
Acomys        61 LKQGASPNVQDASGTSPVHDAARTGFLDTLKVLVEHGADVNALDSTGSLPIHLAIREGHS
Mus           61 LKQGASPNVQDASGTSPVHDAARTGFLDTLKVLVEHGADVNALDSTGSLPIHLAIREGHS
Oryctolagus   61 LKQGASPNVQDASGTAPAHDAARTGFLDTLKVLVDHGADVNVPDGAGALPIHLAVREGHA

Rattus       121 SVVSFLAPESDLHHKDASGLTPLELARQRGAQNLMDILQSHMMIPM
Acomys       121 SVVSFLAPESDLYHRDAAGLTPLELARQRGAHHLADILQRHMVIPV
Mus          121 SVVSFLAPESDLHHRDASGLTPLELARQRGAQNLMDILQGHMMIPM
Oryctolagus  121 AVVSFLAAESDLQHRDARGLTPLELARQRGARDLLDILQRHAGAPL
```

Supplementary Information

(c) <u>CDKN1A (p21)</u>

```
Rattus         1 MSDPGDVR-PVPHRSKVCRRLFGPVDSEQLSRDCDALMASCLQEARERWNFDFATETPLE
Acomys         1 MSDHRDVRPPPPHRSKVCRRLFGPVDSEQLRRDGDALMASCLQEARERWNFDFVTETPLE
Mus            1 MSNLGDVR-PVPHRSKVCRCLFGPVDSEQLRRDCDALMAGCLQEARERWNFDFVTETPLE
Oryctolagus    1 MSQPSDEAPQPPHRSKACRRLFGPVDSEQLRRDCDALMADCLQEARERWNFDFVTETPLE

Rattus        60 GNYVWERVRSPGLPKIYLSPGSRRRDDLGGDKRPSTSSALLQGPGPAPEDHVALSLSCTL
Acomys        61 GNYVWERVGGLGLPKLYLSPGSRGRDDLGGDKRPGTSSALLQGPAPEDHVALSLSCTLVS
Mus           60 GNFVWERVRSLGLPKVYLSPGSRSRDDLGGDKRPSTSSALLQGPAPEDHVALSLSCTLVS
Oryctolagus   61 GNFAWERVRGLGLPKLYLAPGPRGGREDPAGCKRPSTSATLLPAAQEDHVDLSLTCTLVP

Rattus       120 VSHAPERPEDSPGGTGTSQGRKRRQTSLTDFYHSKRRLVFCKRKP
Acomys       121 HAPERPEDSPGGLGTSQGRKRRQTSLTDFYHSKRRLVFCKRKP--
Mus          120 ERPEDSPGGPGTSQGRKRRQTSLTDFYHSKRRLVFCKRKP-----
Oryctolagus  121 RSPERPEESPGGPGTSQGRKRRRQTSLTDFYHSKRRLVFSKRKP-
```

Supplementary Information

(d) P53

```
Rattus          1 -------------MEDSQSDMSIELPLSQETFSCLWKLLPPDDILPTTATG---------
Acomys          1 MKTGSLAGFLDTTMEEPQLDLSIEPPLSQETFSDLWKLLPPKDLSNLLPSDSVEDLPQSN
Mus             1 ----------MTAMEESQSDISLELPLSQETFSGLWKLLPPEDILP--------------
Oryctolagus     1 -------------MEESQSDLSIEPPLSQETFSDLWKLLPENNLLTTSLN----------

Rattus         39 -----------------------------SPNSMEDLFLPQDVAELLEGPEEALQVS-AP
Acomys         61 LLPSDSVEDLPQSNLLPSDSVEDLPQSNLLPSDSVEDLLLFPDVNWLDGLGEAAPMAAAP
Mus            37 -----------------------------SPHCMDDLLLPQDVEEFFEGPSEALRVSGAP
Oryctolagus    38 ------------------------------PPVDDLLSAEDVANWLNEDPEEGLRVPAAP

Rattus         69 AAQEPGTEAPAPVAPASATPWPLSSSVPSQKTYQGNYGFHLGFLQSGTAKSVMCTYSISL
Acomys        121 LAEDPVTEAPAPVAPAPTTPWPLSSFVPSQKTFQGSYGFHLRFLHSGTAKSVTCTYSPSL
Mus            68 AAQDPVTETPGPVAPAPATPWPLSSFVPSQKTYQGNYGFHLGFLQSGTAKSVMCTYSPPL
Oryctolagus    68 APEAPAPAAPALAAPAPATSWPLSSSVPSQKTYHGNYGFRLGFLHSGTAKSVTCTYSPCL

Rattus        129 NKLFCQLAKTCPVQLWVTSTPPPGTRVRAMAIYKKSQHMTEVVRRCPHHERCSDGDGLAP
Acomys        181 NKLFCQLAKTCPVQLWVSSTPPPGTRVRAMAIYKNSQHMTEVVRRCPHHERCSDGDGLAP
Mus           128 NKLFCQLAKTCPVQLWVSATPPAGSRVRAMAIYKKSQHMTEVVRRCPHHERCSDGDGLAP
Oryctolagus   128 NKLFCQLAKTCPVQLWVDSTPPPGTRVRAMAIYKKSQHMTEVVRRCPHHERCSDSDGLAP

Rattus        189 PQHLIRVEGNPYAEYLDDRQTFRHSVVVPYEPPEVGSDYTTIHYKYMCNSSCMGGMNRRP
Acomys        241 PQHLIRVEGNLHAEYVDDRQTFRHSVVVPYESPEQGSDCTTIHYNYMCNSSCMGGMNRRP
Mus           188 PQHLIRVEGNLYPEYLEDRQTFRHSVVVPYEPPEAGSEYTTIHYKYMCNSSCMGGMNRRP
Oryctolagus   188 PQHLIRVEGNLRAEYLDDRNTFRHSVVVPYEPPEVGSDCTTIHYNYMCNSSCMGGMNRRP

Rattus        249 ILTIITLEDSSGNLLGRDSFEVRVCACPGRDRRTEEENFRKKEEHCPELPPGSAKRALPT
Acomys        301 ILTIITLEDASGNLLGRSSFEVRICACPGRDRRTEEENFRKKREGCPELPQGSHKRALPT
Mus           248 ILTIITLEDSSGNLLGRDSFEVRVCACPGRDRRTEEENFRKKEVLCPELPPGSAKRALPT
Oryctolagus   248 ILTIITLEDSSGNLLGRNSFEVRVCACPGRDRRTEEENFRKKGEPCPELPPGSSKRALPT

Rattus        309 STSSSPQQKKKPLDGEYFTLKIRGRERFEMFRELNEALELKDARAAEESGDSRAHSSYPK
Acomys        361 NGNTSPQPKRKPLDGEYFTLKIWGRRRFELFRELNEALELKDAHAAAAAAAAAAEESGD
Mus           308 CTSASPPQKKKPLDGEYFTLKIRGRKRFEMFRELNEALELKDAHATEESGDSRAHSSYLK
Oryctolagus   308 TTTDSSPQTKKKPLDGEYFILKIRGRERFEMFRELNEALELKDAQAEKEPGGSRAHSSYL

Rattus        369 TKKGQSTSRHKKPMIKKVGPDSD---------
Acomys        421 SGPHSSILKTKKDKFTSPRKSLMIKKKKPDSD
Mus           368 TKKGQSTSRHKKTMVKKVGPDSD---------
Oryctolagus   368 KAKKGQSTSRHKKPMFKREGPDSD--------
```

Supplementary Information

(e)

**Percent identity matrix score to *Mus* across all species**			
**Protein name**	***Acomys***	***Rattus***	***Oryctolagus***
**p16**	60.47	77.35	63.51
**p19**	95.18	97.59	84.33
**p21**	87.42	89.30	71.69
**p53**	72.05	86.66	77.43

(f)

**Percent identity matrix score to *Mus* across all species in target region**			
**Target protein**	***Acomys***	***Rattus***	***Oryctolagus***
**p16**	66.67	79.79	66.30
**p19**	90.32	96.77	77.42

Protein alignments across all species for panel of senescent markers. (a-d) The fasta sequences of p16, p19, p21 and p53 were aligned through the multiple sequence alignment tool-clustal omega. **(e-f)** The percent identity matrix score was calculated to show the sequence similarities across all species. Amino acid target sequences for p16 and p19 are indicated in red.

Supplementary Information

Supplementary Tables

(SE = Standard Error, DF = Degree of Freedom, SS=Sum of Squares, MS= Mean Square)

Supplementary Table 1. ANOVA table and Tukey-HSD post-hoc comparisons to test for differences in percent EdU+ cells at 20% O_2 at passage 2 (P2) for *Acomys*, *Mus*, *Rattus* and *Oryctolagus* fibroblasts. *n* = 4/species.

**Source**	**DF**	**SS**	**MS**	***F* Ratio**	***P*-value**
Model	3	1.8136401	0.604547	143.3982	<.0001*
Error	12	0.0505903	0.004216		
C. Total	15	1.8642304			

**Species**	**-Species**	**SE**	***t*-ratio**	***P*-value**
Acomys	*Mus*	0.0459122	13.28	<.0001*
Acomys	*Oryctolagus*	0.0459122	-4.87	0.0019*
Acomys	*Rattus*	0.0459122	-4.38	0.0043*
Mus	*Oryctolagus*	0.0459122	-18.15	<.0001*
Mus	*Rattus*	0.0459122	-17.66	<.0001*
Oryctolagus	*Rattus*	0.0459122	0.49	**0.9598**

Supplementary Information

Supplementary Table 2. One-way ANOVA table and Tukey-HSD post-hoc comparisons to test for differences in percent EdU+ cells at 3% O_2 at passage 2 (P2) for *Acomys*, *Mus*, *Rattus* and *Oryctolagus* fibroblasts. *n* = 4/species

**Source**	**DF**	**SS**	**MS**	***F* Ratio**	***P*-value**
Model	3	0.13588278	0.045294	17.3085	0.0001*
Error	12	0.03140255	0.002617		
C. Total	15	0.16728532			

**Species**	**-Species**	**SE**	***t*-ratio**	***P*-value**
Acomys	*Mus*	0.0361724	6.12	0.0003*
Acomys	*Oryctolagus*	0.0361724	-0.17	**0.9983**
Acomys	*Rattus*	0.0361724	1.37	**0.5382**
Mus	*Oryctolagus*	0.0361724	-6.29	0.0002*
Mus	*Rattus*	0.0361724	-4.75	0.0023*
Oryctolagus	*Rattus*	0.0361724	1.54	**0.4469**

Supplementary Information

Supplementary Table 3. Two-way repeated measures ANOVA table and effect test followed by Tukey-HSD post-hoc comparisons of different passages of *Acomys*, *Mus*, *Rattus* and *Oryctolagus* fibroblasts to test for differences in percent SA-βgal+ cells at 3% O_2 across passage. n = 5 (*Acomys, Mus*) and n=4 (*Rattus*, *Oryctolagus*).

**Source**	**DF**	**SS**	**MS**	***F* Ratio**	***P*-value**
Model	35	11.676187	0.333605	240.5825	<.0001*
Error	118	0.163625	0.001387		
C. Total	153	11.839812			

**Source**	**DF**	**SS**	**MS**	***F* Ratio**	***P*-value**	**Notes**
Species	2	4.2081889	2.104094	1517.387	<.0001*	Lost DFs
Passages	5	1.2188249	0.243765	175.7934	<.0001*	Lost DFs
Species*Passages	23	0.9566262	0.041592	29.9948	<.0001*	Lost DFs

**Species**	**Passages**	**Species**	**Passages**	**SE**	***t*-ratio**	***P*-value**
Acomys	P1	*Mus*	P1	0.0235513	-5.26	0.0004*
Acomys	P1	*Rattus*	P1	0.0249799	7.27	<.0001*
Acomys	P1	*Oryctolagus*	P1	0.0249799	10.43	<.0001*
Mus	P1	*Rattus*	P1	0.0249799	12.23	<.0001*
Mus	P1	*Oryctolagus*	P1	0.0249799	15.39	<.0001*
Rattus	P1	*Oryctolagus*	P1	0.0263311	-2.99	**0.5322**
Acomys	P13	*Mus*	P13	0.0271947	-23.81	<.0001*
Acomys	P13	*Rattus*	P13	0.0249799	7.43	<.0001*
Acomys	P13	*Oryctolagus*	P13	0.0249799	19.60	<.0001*
Mus	P13	*Rattus*	P13	0.0284409	29.29	<.0001*
Mus	P13	*Oryctolagus*	P13	0.0284409	39.98	<.0001*
Rattus	P13	*Oryctolagus*	P13	0.0263311	-11.54	<.0001*
Acomys	P25	*Rattus*	P25	0.0284409	7.44	<.0001*
Acomys	P25	*Oryctolagus*	P25	0.0284409	22.51	<.0001*
Rattus	P25	*Oryctolagus*	P25	0.0263311	-16.28	<.0001*

Supplementary Information

Supplementary Table 4. One-way ANOVA tables to test for differences in percent γ-H2AX+, p16+, p19+, p21+ and p53+ cells at 20% O_2 at passage 2 (P2) for *Acomys* and *Mus* fibroblasts. *n* = 3/species.

γ-H2AX+ cells:

Source	DF	SS	MS	*F* Ratio	*P*-value
Species	1	0.39334422	0.393344	122.89	0.0004*
Error	4	0.01280314	0.003201		
C. Total	5	0.40614736			

p16+ cells:

Source	DF	SS	MS	*F* Ratio	*P*-value
Species	1	0.59012946	0.590129	1344.405	<.0001*
Error	4	0.00175581	0.000439		
C. Total	5	0.59188527			

p19+ cells:

Source	DF	SS	MS	*F* Ratio	*P*-value
Species	1	0.59915874	0.599159	531.8164	<.0001*
Error	4	0.00450651	0.001127		
C. Total	5	0.60366525			

p21+ cells:

Source	DF	SS	MS	*F* Ratio	*P*-value
Species	1	0.87138249	0.871382	483.2017	<.0001*
Error	4	0.00721341	0.001803		
C. Total	5	0.8785959			

p53+ cells:

Source	DF	SS	MS	*F* Ratio	*P*-value
Species	1	0.91160233	0.911602	676.4388	<.0001*
Error	4	0.0053906	0.001348		
C. Total	5	0.91699293			

Supplementary Information

Supplementary Table 5. Two-way ANOVA table and effect test followed by Tukey-HSD post-hoc comparisons of passage 2 (P2) *Acomys* and *Mus* H_2O_2 treated fibroblasts cultured for 24hrs to test for differences in percent EdU+ cells at 3% O_2. n = 4/species

**Source**	**DF**	**SS**	**MS**	***F* Ratio**	***P*-value**
Model	7	0.50795239	0.072565	76.9827	<.0001*
Error	24	0.02262264	0.000943		
C. Total	31	0.53057503			

**Source**	**DF**	**SS**	**MS**	***F* Ratio**	***P*-value**
Species	1	0.49929011	0.4992901	529.689	<.0001*
Concentration	3	0.00846757	0.0028225	2.9944	**0.0507**
Species*Concentration	3	0.00019471	0.0000649	0.0689	**0.976**

**Species**	**Concentration**	**Species**	**Concentration**	**DF**	**SE**	***t*-ratio**	***P*-value**
Acomys	0μM H_2O_2	*Acomys*	75μM H_2O_2	24	0.0217096	0.73	**0.9952**
Acomys	0μM H_2O_2	*Acomys*	150μM H_2O_2	24	0.0217096	0.90	**0.9837**
Acomys	0μM H_2O_2	*Acomys*	300μM H_2O_2	24	0.0217096	1.79	**0.6323**
Mus	0μM H_2O_2	*Mus*	75μM H_2O_2	24	0.0217096	1.08	**0.9558**
Mus	0μM H_2O_2	*Mus*	150μM H_2O_2	24	0.0217096	1.26	**0.9039**
Mus	0μM H_2O_2	*Mus*	300μM H_2O_2	24	0.0217096	2.43	**0.2721**

Supplementary Information

Supplementary Table 6. Two-way ANOVA table and effect test followed by Tukey-HSD post-hoc comparisons of passage 2 (P2) *Acomys* and *Mus* H_2O_2 treated fibroblasts cultured for 48hrs to test for differences in percent EdU+ cells at 3% O_2. n = 4/species

**Source**	**DF**	**SS**	**MS**	***F* Ratio**	***P*-value**
Model	7	0.63440329	0.090629	63.3481	<.0001*
Error	24	0.03433563	0.001431		
C. Total	31	0.66873892			

**Source**	**DF**	**SS**	**MS**	***F* Ratio**	***P*-value**
Species	1	0.51365713	0.5136571	359.0373	<.0001*
Concentration	3	0.1010219	0.033674	23.5375	<.0001*
Species*Concentration	3	0.01972426	0.0065748	4.5956	0.0112*

**Species**	**Concentration**	**Species**	**Concentration**	**DF**	**SE**	***t*-ratio**	***P*-value**
Acomys	0μM H_2O_2	*Acomys*	75μM H_2O_2	24	0.0267456	0.82	**0.9899**
Acomys	0μM H_2O_2	*Acomys*	150μM H_2O_2	24	0.0267456	1.23	**0.9160**
Acomys	0μM H_2O_2	*Acomys*	300μM H_2O_2	24	0.0267456	3.32	0.0493*
Mus	0μM H_2O_2	*Mus*	75μM H_2O_2	24	0.0267456	1.83	**0.6103**
Mus	0μM H_2O_2	*Mus*	150μM H_2O_2	24	0.0267456	7.94	0.0019*
Mus	0μM H_2O_2	*Mus*	300μM H_2O_2	24	0.0267456	2.43	<.0001*

Supplementary Information

Supplementary Table 7. Two-way ANOVA table and effect test followed by Tukey-HSD post-hoc comparisons of passage 2 (P2) *Acomys* and *Mus* H_2O_2 treated fibroblasts cultured for 24hrs to test for differences in percent SAβ-gal+ cells at 3% O_2. $n = 4$/species.

**Source**	**DF**	**SS**	**MS**	***F* Ratio**	***P*-value**
Model	7	0.00157176	0.000225	1.1424	**0.3708**
Error	24	0.00471734	0.000197		
C. Total	31	0.00628911			

**Source**	**DF**	**SS**	**MS**	***F* Ratio**	***P*-value**
Species	1	0.00000419	0.0000042	0.0213	**0.8851**
Concentration	3	0.00108725	0.0003624	1.8438	**0.1662**
Species*Concentration	3	0.00048032	0.0001601	0.8146	**0.4984**

**Species**	**Concentration**	**Species**	**Concentration**	**DF**	**SE**	***t-ratio***	***P*-value**
Acomys	0μM H_2O_2	*Acomys*	75μM H_2O_2	24	0.0099135	-0.36	**1.0000**
Acomys	0μM H_2O_2	*Acomys*	150μM H_2O_2	24	0.0099135	-1.22	**0.9174**
Acomys	0μM H_2O_2	*Acomys*	300μM H_2O_2	24	0.0099135	-0.09	**1.0000**
Mus	0μM H_2O_2	*Mus*	75μM H_2O_2	24	0.0099135	-2.41	**0.2817**
Mus	0μM H_2O_2	*Mus*	150μM H_2O_2	24	0.0099135	-1.54	**0.7789**
Mus	0μM H_2O_2	*Mus*	300μM H_2O_2	24	0.0099135	-0.98	**0.9725**

Supplementary Information

Supplementary Table 8. Two-way ANOVA table and effect test followed by Tukey-HSD post-hoc comparisons of passage 2 (P2) *Acomys* and *Mus* H_2O_2 treated fibroblasts cultured for 48hrs to test for differences in percent SAβ-gal+ cells at 3% O_2. n = 4/species

**Source**	**DF**	**SS**	**MS**	***F* Ratio**	***P*-value**
Model	7	0.12711831	0.01816	223.0515	<.0001*
Error	24	0.00195396	0.000081		
C. Total	31	0.12907227			

**Source**	**DF**	**SS**	**MS**	***F* Ratio**	***P*-value**
Species	1	0.08094173	0.0809417	994.1859	<.0001*
Concentration	3	0.03001148	0.0100038	122.8743	<.0001*
Species*Concentration	3	0.0161651	0.0053884	66.1839	<.0001*

**Species**	**Concentration**	**Species**	**Concentration**	**DF**	**SE**	***t-ratio***	***P*-value**
Acomys	0μM H_2O_2	*Acomys*	75μM H_2O_2	24	0.0063802	-0.85	**0.9875**
Acomys	0μM H_2O_2	*Acomys*	150μM H_2O_2	24	0.0063802	-1.33	**0.8790**
Acomys	0μM H_2O_2	*Acomys*	300μM H_2O_2	24	0.0063802	-3.75	0.0189*
Mus	0μM H_2O_2	*Mus*	75μM H_2O_2	24	0.0063802	-7.42	<.0001*
Mus	0μM H_2O_2	*Mus*	150μM H_2O_2	24	0.0063802	-15.83	<.0001*
Mus	0μM H_2O_2	*Mus*	300μM H_2O_2	24	0.0063802	-21.91	<.0001*

Supplementary Information

Supplementary Table 9. Two-way ANOVA table and effect test followed by Tukey-HSD post-hoc comparisons of passage 2 (P2) *Acomys* and *Mus* H_2O_2 treated fibroblasts cultured for 24hrs to test for differences in percent γH2AX + at 3% O_2. *n* = 4/species

**Source**	**DF**	**SS**	**MS**	***F* Ratio**	***P*-value**
Model	7	0.93711498	0.133874	168.5012	<.0001*
Error	24	0.01906791	0.000794		
C. Total	31	0.95618289			

**Source**	**DF**	**SS**	**MS**	***F* Ratio**	***P*-value**
Species	1	0.75181847	0.7518185	946.2832	<.0001*
Concentration	3	0.09080154	0.0302672	38.0961	<.0001*
Species*Concentration	3	0.09449497	0.0314983	39.6457	<.0001*

**Species**	**Concentration**	**Species**	**Concentration**	**DF**	**SE**	***t-ratio***	***P*-value**
Acomys	0μM H_2O_2	*Acomys*	75μM H_2O_2	24	0.0199311	-0.31	***1.0000***
Acomys	0μM H_2O_2	*Acomys*	150μM H_2O_2	24	0.0199311	0.19	***1.0000***
Acomys	0μM H_2O_2	*Acomys*	300μM H_2O_2	24	0.0199311	0.08	***1.0000***
Mus	0μM H_2O_2	*Mus*	75μM H_2O_2	24	0.0199311	-3.05	***0.0870***
Mus	0μM H_2O_2	*Mus*	150μM H_2O_2	24	0.0199311	-3.83	0.0155*
Mus	0μM H_2O_2	*Mus*	300μM H_2O_2	24	0.0199311	-14.31	<.0001*

Supplementary Information

Supplementary Table 10. Two-way ANOVA table and effect test followed by Tukey-HSD post-hoc comparisons of passage 2 (P2) *Acomys* and *Mus* H_2O_2 treated fibroblasts cultured for 48hrs to test for differences in percent γH2AX+ cells at 3% O_2. n = 4/species

**Source**	**DF**	**SS**	**MS**	***F* Ratio**	***P*-value**
Model	7	0.01040014	0.001486	377.375	<.0001*
Error	24	0.00009449	3.94E-06		
C. Total	31	0.01049462			

**Source**	**DF**	**SS**	**MS**	***F* Ratio**	***P*-value**
Species	1	0.00901419	0.0090142	2289.596	<.0001*
Concentration	3	0.00095015	0.0003167	80.4456	<.0001*
Species*Concentration	3	0.0004358	0.0001453	36.8974	<.0001*

**Species**	**Concentration**	**Species**	**Concentration**	**DF**	**SE**	***t-ratio***	***P*-value**
Acomys	0μM H_2O_2	*Acomys*	75μM H_2O_2	24	0.0014030	-0.56	**0.9990**
Acomys	0μM H_2O_2	*Acomys*	150μM H_2O_2	24	0.0014030	-0.98	**0.9734**
Acomys	0μM H_2O_2	*Acomys*	300μM H_2O_2	24	0.0014030	-3.55	0.0296*
Mus	0μM H_2O_2	*Mus*	75μM H_2O_2	24	0.0014030	-4.96	0.0010*
Mus	0μM H_2O_2	*Mus*	150μM H_2O_2	24	0.0014030	-11.01	<.0001*
Mus	0μM H_2O_2	*Mus*	300μM H_2O_2	24	0.0014030	-17.31	<.0001*

Supplementary Information

Supplementary Table 11. Two-way ANOVA table and effect test followed by Tukey-HSD post-hoc comparisons of passage 2 (P2) *Acomys* and *Mus* H_2O_2 treated fibroblasts cultured for 48hrs to test for differences in percent p16+ cells at 3% O_2. *n* = 4/species

**Source**	**DF**	**SS**	**MS**	***F* Ratio**	***P*-value**
Model	7	3.0718042	0.438829	783.3681	<.0001*
Error	24	0.0134444	0.00056		
C. Total	31	3.0852486			

**Source**	**DF**	**SS**	**MS**	***F* Ratio**	***P*-value**
Species	1	2.7740331	2.774033	4952.016	<.0001*
Concentration	3	0.1560128	0.052004	92.8345	<.0001*
Species*Concentration	3	0.1417583	0.047253	84.3524	<.0001*

**Species**	**Concentration**	**Species**	**Concentration**	**DF**	**SE**	***t-ratio***	***P*-value**
Acomys	0μM H_2O_2	*Acomys*	75μM H_2O_2	24	0.0167359	-0.10	**1.0000**
Acomys	0μM H_2O_2	*Acomys*	150μM H_2O_2	24	0.0167359	-0.42	**0.9999**
Acomys	0μM H_2O_2	*Acomys*	300μM H_2O_2	24	0.0167359	-0.49	**0.9996**
Mus	0μM H_2O_2	*Mus*	75μM H_2O_2	24	0.0167359	-7.29	<.0001*
Mus	0μM H_2O_2	*Mus*	150μM H_2O_2	24	0.0167359	-13.24	<.0001*
Mus	0μM H_2O_2	*Mus*	300μM H_2O_2	24	0.0167359	-22.24	<.0001*

Supplementary Information

Supplementary Table 12. Two-way ANOVA table and effect test followed by Tukey-HSD post-hoc comparisons of passage 2 (P2) *Acomys* and *Mus* H_2O_2 treated fibroblasts which were fixed after 48 hrs to test for differences in percent p19+ cells at 3% O_2. n = 4/species.

**Source**	**DF**	**SS**	**MS**	***F* Ratio**	***P*-value**
Model	7	3.6133041	0.516186	1094.501	<.0001*
Error	24	0.0113188	0.000472		
C. Total	31	3.6246229			

**Source**	**DF**	**SS**	**MS**	***F* Ratio**	***P*-value**
Species	1	3.1955264	3.195526	6775.668	<.0001*
Concentration	3	0.231747	0.077249	163.7958	<.0001*
Species*Concentration	3	0.1860307	0.06201	131.484	<.0001*

**Species**	**Concentration**	**Species**	**Concentration**	**DF**	**SE**	***t-ratio***	***P*-value**
Acomys	0µM H_2O_2	*Acomys*	75µM H_2O_2	24	0.0153561	-0.38	**0.9999**
Acomys	0µM H_2O_2	*Acomys*	150µM H_2O_2	24	0.0153561	-1.20	**0.9248**
Acomys	0µM H_2O_2	*Acomys*	300µM H_2O_2	24	0.0153561	-1.46	**0.8184**
Mus	0µM H_2O_2	*Mus*	75µM H_2O_2	24	0.0153561	-9.14	<.0001*
Mus	0µM H_2O_2	*Mus*	150µM H_2O_2	24	0.0153561	-17.64	<.0001*
Mus	0µM H_2O_2	*Mus*	300µM H_2O_2	24	0.0153561	-28.45	<.0001*

Supplementary Information

Supplementary Table 13. Two-way ANOVA table and effect test followed by Tukey-HSD post-hoc comparisons of passage 2 (P2) *Acomys* and *Mus* H_2O_2 treated fibroblasts cultured for 48hrs to test for differences in percent p21+ cells at 3% O_2. n = 4/species.

**Source**	**DF**	**SS**	**MS**	***F* Ratio**	***P*-value**
Model	7	2.8679554	0.409708	526.5525	<.0001*
Error	24	0.0186743	0.000778		
C. Total	31	2.8866297			

**Source**	**DF**	**SS**	**MS**	***F* Ratio**	***P*-value**
Species	1	2.5203031	2.520303	3239.068	<.0001*
Concentration	3	0.1804059	0.060135	77.2853	<.0001*
Species*Concentration	3	0.1672464	0.055749	71.6478	<.0001*

**Species**	**Concentration**	**Species**	**Concentration**	**DF**	**SE**	***t-ratio***	***P*-value**
Acomys	0μM H_2O_2	*Acomys*	75μM H_2O_2	24	0.0197243	0.05	**1.0000**
Acomys	0μM H_2O_2	*Acomys*	150μM H_2O_2	24	0.0197243	-0.29	**1.0000**
Acomys	0μM H_2O_2	*Acomys*	300μM H_2O_2	24	0.0197243	-0.43	**0.9998**
Mus	0μM H_2O_2	*Mus*	75μM H_2O_2	24	0.0197243	-13.35	<.0001*
Mus	0μM H_2O_2	*Mus*	150μM H_2O_2	24	0.0197243	-15.93	<.0001*
Mus	0μM H_2O_2	*Mus*	300μM H_2O_2	24	0.0197243	-19.89	<.0001*

Supplementary Information

Supplementary Table 14. Two-way ANOVA table and effect test followed by Tukey-HSD post-hoc comparisons of passage 2 (P2) *Acomys* and *Mus* H_2O_2 treated fibroblasts cultured for 48hrs to test for differences in percent p53+ cells at 3% O_2. $n = 4$/species

**Source**	**DF**	**SS**	**MS**	***F* Ratio**	***P*-value**
Model	7	2.6094404	0.372777	399.1625	<.0001*
Error	24	0.0224136	0.000934		
C. Total	31	2.631854			

**Source**	**DF**	**SS**	**MS**	***F* Ratio**	***P*-value**
Species	1	2.3598669	2.359867	2526.899	<.0001*
Concentration	3	0.1296007	0.0432	46.2579	<.0001*
Species*Concentration	3	0.1199728	0.039991	42.8215	<.0001*

**Species**	**Concentration**	**Species**	**Concentration**	**DF**	**SE**	***t-ratio***	***P*-value**
Acomys	0μM H_2O_2	*Acomys*	75μM H_2O_2	24	0.0216090	-0.06	**1.0000**
Acomys	0μM H_2O_2	*Acomys*	150μM H_2O_2	24	0.0216090	-0.31	**1.0000**
Acomys	0μM H_2O_2	*Acomys*	300μM H_2O_2	24	0.0216090	-0.30	**1.0000**
Mus	0μM H_2O_2	*Mus*	75μM H_2O_2	24	0.0216090	-10.60	<.0001*
Mus	0μM H_2O_2	*Mus*	150μM H_2O_2	24	0.0216090	-11.74	<.0001*
Mus	0μM H_2O_2	*Mus*	300μM H_2O_2	24	0.0216090	-15.59	<.0001*

Supplementary Information

Supplementary Table 15. Two-way ANOVA table and effect test followed by Tukey-HSD post-hoc comparisons of passage 2 (P2) *Acomys*, *Mus*, *Rattus* and *Oryctolagus* H_2O_2 treated fibroblasts cultured for 48hrs to test for differences in percent EdU+ cells at 3% O_2. *n* = 4/species

**Source**	**DF**	**SS**	**MS**	***F* Ratio**	***P*-value**
Model	15	1.3015089	0.086767	96.8033	<.0001*
Error	48	0.0430236	0.000896		
C. Total	63	1.3445325			

**Source**	**DF**	**SS**	**MS**	***F* Ratio**	***P*-value**
Species	3	1.0071227	0.3357076	374.5375	<.0001*
Concentration	3	0.2010950	0.0670317	74.7849	<.0001*
Species*Concentration	9	0.0932912	0.0103657	11.5646	<.0001*

**Species**	**Concentration**	**Species**	**Concentration**	**DF**	**SE**	***t-ratio***	***P*-value**
Acomys	0μM H_2O_2	*Acomys*	75μM H_2O_2	48	0.0211699	1.04	**0.9995**
Acomys	0μM H_2O_2	*Acomys*	150μM H_2O_2	48	0.0211699	1.55	**0.9699**
Acomys	0μM H_2O_2	*Acomys*	300μM H_2O_2	48	0.0211699	4.19	0.0099*
Mus	0μM H_2O_2	*Mus*	75μM H_2O_2	48	0.0211699	2.31	**0.6214**
Mus	0μM H_2O_2	*Mus*	150μM H_2O_2	48	0.0211699	5.95	<.0001*
Mus	0μM H_2O_2	*Mus*	300μM H_2O_2	48	0.0211699	10.04	<.0001*
Rattus	0μM H_2O_2	*Rattus*	75μM H_2O_2	48	0.0211699	3.20	**0.1364**
Rattus	0μM H_2O_2	*Rattus*	150μM H_2O_2	48	0.0211699	8.25	<.0001*
Rattus	0μM H_2O_2	*Rattus*	300μM H_2O_2	48	0.0211699	12.88	<.0001*
Oryctolagus	0μM H_2O_2	*Oryctolagus*	75μM H_2O_2	48	0.0211699	0.46	**1.0000**
Oryctolagus	0μM H_2O_2	*Oryctolagus*	150μM H_2O_2	48	0.0211699	0.86	**0.9999**
Oryctolagus	0μM H_2O_2	*Oryctolagus*	300μM H_2O_2	48	0.0211699	1.09	**0.9991**

Supplementary Information

Supplementary Table 16. Two-way ANOVA table and effect test followed by Tukey-HSD post-hoc comparisons of passage 2 (P2) *Acomys*, *Mus*, *Rattus* and *Oryctolagus* H_2O_2 treated fibroblasts cultured for 48hrs to test for differences in percent γ-H2AX+ cells at 3% O_2. n = 4/species

**Source**	**DF**	**SS**	**MS**	***F* Ratio**	***P*-value**
Model	15	2.9637590	0.197584	458.4376	<.0001*
Error	48	0.0206877	0.000431		
C. Total	63	2.9844467			

**Source**	**DF**	**SS**	**MS**	***F* Ratio**	***P*-value**
Species	3	2.5654649	0.8551550	1984.1450	<.0001*
Concentration	3	0.2291150	0.0763717	177.1989	<.0001*
Species*Concentration	9	0.1691791	0.0187977	43.6147	<.0001*

**Species**	**Concentration**	**Species**	**Concentration**	**DF**	**SE**	***t-ratio***	***P*-value**
Acomys	0μM H_2O_2	*Acomys*	75μM H_2O_2	48	0.0146798	-0.56	**1.0000**
Acomys	0μM H_2O_2	*Acomys*	150μM H_2O_2	48	0.0146798	-0.98	**0.9997**
Acomys	0μM H_2O_2	*Acomys*	300μM H_2O_2	48	0.0146798	-2.26	**0.6524**
Mus	0μM H_2O_2	*Mus*	75μM H_2O_2	48	0.0146798	-5.71	<.0001*
Mus	0μM H_2O_2	*Mus*	150μM H_2O_2	48	0.0146798	-13.16	<.0001*
Mus	0μM H_2O_2	*Mus*	300μM H_2O_2	48	0.0146798	-21.79	<.0001*
Rattus	0μM H_2O_2	*Rattus*	75μM H_2O_2	48	0.0146798	-7.23	<.0001*
Rattus	0μM H_2O_2	*Rattus*	150μM H_2O_2	48	0.0146798	-13.71	<.0001*
Rattus	0μM H_2O_2	*Rattus*	300μM H_2O_2	48	0.0146798	-18.33	<.0001*
Oryctolagus	0μM H_2O_2	*Oryctolagus*	75μM H_2O_2	48	0.0146798	-0.14	**1.0000**
Oryctolagus	0μM H_2O_2	*Oryctolagus*	150μM H_2O_2	48	0.0146798	-0.15	**1.0000**
Oryctolagus	0μM H_2O_2	*Oryctolagus*	300μM H_2O_2	48	0.0146798	-1.42	**0.9862**

Supplementary Information

Supplementary Table 17. Two-way ANOVA table and effect test followed by Tukey-HSD post-hoc comparisons of passage 2 (P2) *Acomys*, *Mus*, *Rattus* and *Oryctolagus* H_2O_2 treated fibroblasts cultured for 48hrs to test for differences in percent SAβ-gal+ cells at 3% O_2. n = 4/species

**Source**	**DF**	**SS**	**MS**	***F* Ratio**	***P*-value**
Model	15	11673.223	778.215	400.8695	<.0001*
Error	48	93.183	1.941		
C. Total	63	11766.406			

**Source**	**DF**	**SS**	**MS**	***F* Ratio**	***P*-value**
Species	3	10007.054	3335.685	1718.2584	<.0001*
Concentration	3	931.108	310.369	159.8756	<.0001*
Species*Concentration	9	735.061	81.673	42.0712	<.0001*

**Species**	**Concentration**	**Species**	**Concentration**	**DF**	**SE**	***t-ratio***	***P*-value**
Acomys	0μM H_2O_2	*Acomys*	75μM H_2O_2	48	0.9852201	-1.47	**0.9813**
Acomys	0μM H_2O_2	*Acomys*	150μM H_2O_2	48	0.9852201	-2.38	**0.5703**
Acomys	0μM H_2O_2	*Acomys*	300μM H_2O_2	48	0.9852201	-3.45	**0.0757**
Mus	0μM H_2O_2	*Mus*	75μM H_2O_2	48	0.9852201	-4.49	0.0040*
Mus	0μM H_2O_2	*Mus*	150μM H_2O_2	48	0.9852201	-9.75	<.0001*
Mus	0μM H_2O_2	*Mus*	300μM H_2O_2	48	0.9852201	-13.63	<.0001*
Rattus	0μM H_2O_2	*Rattus*	75μM H_2O_2	48	0.9852201	-7.05	<.0001*
Rattus	0μM H_2O_2	*Rattus*	150μM H_2O_2	48	0.9852201	-14.10	<.0001*
Rattus	0μM H_2O_2	*Rattus*	300μM H_2O_2	48	0.9852201	-24.03	<.0001*
Oryctolagus	0μM H_2O_2	*Oryctolagus*	75μM H_2O_2	48	0.9852201	-0.43	**1.0000**
Oryctolagus	0μM H_2O_2	*Oryctolagus*	150μM H_2O_2	48	0.9852201	-0.49	**1.0000**
Oryctolagus	0μM H_2O_2	*Oryctolagus*	300μM H_2O_2	48	0.9852201	-0.61	**1.0000**

Supplementary Information

Supplementary Table 18. Two-way ANOVA table and effect test followed by Tukey-HSD post-hoc comparisons of passage 2 (P2) *Acomys*, *Mus*, *Rattus* and *Oryctolagus* H_2O_2 treated fibroblasts cultured for 48hrs to test for differences in percent p21+ cells at 3% O_2. *n* = 4/species

**Source**	**DF**	**SS**	**MS**	***F* Ratio**	***P*-value**
Model	15	5.5800294	0.372002	501.4221	<.0001*
Error	48	0.0356109	0.000742		
C. Total	63	5.6156403			

**Source**	**DF**	**SS**	**MS**	***F* Ratio**	***P*-value**
Species	3	4.8859517	1.628651	2195.2609	<.0001*
Concentration	3	0.3824328	0.127478	171.8273	<.0001*
Species*Concentration	9	0.3116449	0.034627	46.6741	<.0001*

**Species**	**Concentration**	**Species**	**Concentration**	**DF**	**SE**	***t-ratio***	***P*-value**
Acomys	0μM H_2O_2	*Acomys*	75μM H_2O_2	48	0.0192600	0.05	**1.0000**
Acomys	0μM H_2O_2	*Acomys*	150μM H_2O_2	48	0.0192600	-0.29	**1.0000**
Acomys	0μM H_2O_2	*Acomys*	300μM H_2O_2	48	0.0192600	-0.44	**1.0000**
Mus	0μM H_2O_2	*Mus*	75μM H_2O_2	48	0.0192600	-13.67	<.0001*
Mus	0μM H_2O_2	*Mus*	150μM H_2O_2	48	0.0192600	-16.32	<.0001*
Mus	0μM H_2O_2	*Mus*	300μM H_2O_2	48	0.0192600	-20.37	<.0001*
Rattus	0μM H_2O_2	*Rattus*	75μM H_2O_2	48	0.0192600	-11.83	<.0001*
Rattus	0μM H_2O_2	*Rattus*	150μM H_2O_2	48	0.0192600	-16.24	<.0001*
Rattus	0μM H_2O_2	*Rattus*	300μM H_2O_2	48	0.0192600	-20.30	<.0001*
Oryctolagus	0μM H_2O_2	*Oryctolagus*	75μM H_2O_2	48	0.0192600	-0.09	**1.0000**
Oryctolagus	0μM H_2O_2	*Oryctolagus*	150μM H_2O_2	48	0.0192600	-1.61	**0.9593**
Oryctolagus	0μM H_2O_2	*Oryctolagus*	300μM H_2O_2	48	0.0192600	-1.73	**0.9272**

Supplementary Information

Supplementary Table 19. Two-way ANOVA table and effect test followed by Tukey-HSD post-hoc comparisons of passage 2 (P2) *Acomys*, *Mus*, *Rattus* and *Oryctolagus* H_2O_2 treated fibroblasts cultured for 48hrs to test for differences in percent p53+ cells at 3% O_2. n = 4/species

**Source**	**DF**	**SS**	**MS**	***F* Ratio**	***P*-value**
Model	15	5.2533102	0.350221	564.1753	<.0001*
Error	48	0.0297968	0.000621		
C. Total	63	5.2831069			

**Source**	**DF**	**SS**	**MS**	***F* Ratio**	***P*-value**
Species	3	4.6881052	1.562702	2517.3778	<.0001*
Concentration	3	0.3022541	0.100751	162.3018	<.0001*
Species*Concentration	9	0.2629508	0.029217	47.0657	<.0001*

**Species**	**Concentration**	**Species**	**Concentration**	**DF**	**SE**	***t-ratio***	***P*-value**
Acomys	0μM H_2O_2	*Acomys*	75μM H_2O_2	48	0.0176177	-0.08	**1.0000**
Acomys	0μM H_2O_2	*Acomys*	150μM H_2O_2	48	0.0176177	-0.38	**1.0000**
Acomys	0μM H_2O_2	*Acomys*	300μM H_2O_2	48	0.0176177	-0.36	**1.0000**
Mus	0μM H_2O_2	*Mus*	75μM H_2O_2	48	0.0176177	-13.00	<.0001*
Mus	0μM H_2O_2	*Mus*	150μM H_2O_2	48	0.0176177	-14.40	<.0001*
Mus	0μM H_2O_2	*Mus*	300μM H_2O_2	48	0.0176177	-19.12	<.0001*
Rattus	0μM H_2O_2	*Rattus*	75μM H_2O_2	48	0.0176177	-14.16	<.0001*
Rattus	0μM H_2O_2	*Rattus*	150μM H_2O_2	48	0.0176177	-17.35	<.0001*
Rattus	0μM H_2O_2	*Rattus*	300μM H_2O_2	48	0.0176177	-20.99	<.0001*
Oryctolagus	0μM H_2O_2	*Oryctolagus*	75μM H_2O_2	48	0.0176177	-0.27	**1.0000**
Oryctolagus	0μM H_2O_2	*Oryctolagus*	150μM H_2O_2	48	0.0176177	-0.40	**1.0000**
Oryctolagus	0μM H_2O_2	*Oryctolagus*	300μM H_2O_2	48	0.0176177	-1.47	**0.9814**

Supplementary Table 20. Two-way ANOVA table and effect test followed by Tukey-HSD post-hoc comparisons of passage 2 (P2) *Acomys*, *Mus*, *Rattus* and *Oryctolagus* H_2O_2 treated fibroblasts cultured for 48hrs to test for differences in percent p16+ cells at 3% O_2. *n* = 4/species

**Source**	**DF**	**SS**	**MS**	***F* Ratio**	***P*-value**
Model	15	4.3309883	0.288733	757.2077	<.0001*
Error	48	0.0183030	0.000381		
C. Total	63	4.3492913			

**Source**	**DF**	**SS**	**MS**	***F* Ratio**	***P*-value**
Species	3	3.9571017	1.319034	3459.1964	<.0001*
Concentration	3	0.1894554	0.063152	165.6171	<.0001*
Species*Concentration	9	0.1844312	0.020492	53.7417	<.0001*

**Species**	**Concentration**	**Species**	**Concentration**	**DF**	**SE**	***t-ratio***	***P*-value**
Acomys	0μM H_2O_2	*Acomys*	75μM H_2O_2	48	0.0138078	0.47	**1.0000**
Acomys	0μM H_2O_2	*Acomys*	150μM H_2O_2	48	0.0138078	0.08	**1.0000**
Acomys	0μM H_2O_2	*Acomys*	300μM H_2O_2	48	0.0138078	-2.34	**0.5965**
Mus	0μM H_2O_2	*Mus*	75μM H_2O_2	48	0.0138078	-8.84	<.0001*
Mus	0μM H_2O_2	*Mus*	150μM H_2O_2	48	0.0138078	-16.05	<.0001*
Mus	0μM H_2O_2	*Mus*	300μM H_2O_2	48	0.0138078	-26.95	<.0001*
Rattus	0μM H_2O_2	*Rattus*	75μM H_2O_2	48	0.0138078	-6.25	<.0001*
Rattus	0μM H_2O_2	*Rattus*	150μM H_2O_2	48	0.0138078	-9.25	<.0001*
Rattus	0μM H_2O_2	*Rattus*	300μM H_2O_2	48	0.0138078	-13.36	<.0001*
Oryctolagus	0μM H_2O_2	*Oryctolagus*	75μM H_2O_2	48	0.0138078	-0.12	**1.0000**
Oryctolagus	0μM H_2O_2	*Oryctolagus*	150μM H_2O_2	48	0.0138078	-0.42	**1.0000**
Oryctolagus	0μM H_2O_2	*Oryctolagus*	300μM H_2O_2	48	0.0138078	-0.53	**1.0000**

Supplementary Information

Supplementary Table 21. Two-way ANOVA table and effect test followed by Tukey-HSD post-hoc comparisons of passage 2 (P2) *Acomys*, *Mus*, *Rattus* and *Oryctolagus* H_2O_2 treated fibroblasts cultured for 48hrs to test for differences in percent p19+ cells at 3% O_2. *n* = 4/species

**Source**	**DF**	**SS**	**MS**	***F* Ratio**	***P*-value**
Model	15	5.7408201	0.382721	1064.6167	<.0001*
Error	48	0.0172556	0.000359		
C. Total	63	5.7580757			

**Source**	**DF**	**SS**	**MS**	***F* Ratio**	***P*-value**
Species	3	5.0459322	1.681977	4678.7599	<.0001*
Concentration	3	0.3663475	0.122116	339.6899	<.0001*
Species*Concentration	9	0.3285404	0.036504	101.5446	<.0001*

**Species**	**Concentration**	**Species**	**Concentration**	**DF**	**SE**	***t-ratio***	***P*-value**
Acomys	0μM H_2O_2	*Acomys*	75μM H_2O_2	48	0.0134069	-0.44	**1.0000**
Acomys	0μM H_2O_2	*Acomys*	150μM H_2O_2	48	0.0134069	-1.37	**0.9899**
Acomys	0μM H_2O_2	*Acomys*	300μM H_2O_2	48	0.0134069	-1.68	**0.9431**
Mus	0μM H_2O_2	*Mus*	75μM H_2O_2	48	0.0134069	-10.47	<.0001*
Mus	0μM H_2O_2	*Mus*	150μM H_2O_2	48	0.0134069	-20.21	<.0001*
Mus	0μM H_2O_2	*Mus*	300μM H_2O_2	48	0.0134069	-32.59	<.0001*
Rattus	0μM H_2O_2	*Rattus*	75μM H_2O_2	48	0.0134069	-9.43	<.0001*
Rattus	0μM H_2O_2	*Rattus*	150μM H_2O_2	48	0.0134069	-20.07	<.0001*
Rattus	0μM H_2O_2	*Rattus*	300μM H_2O_2	48	0.0134069	-25.48	<.0001*
Oryctolagus	0μM H_2O_2	*Oryctolagus*	75μM H_2O_2	48	0.0134069	-0.18	**1.0000**
Oryctolagus	0μM H_2O_2	*Oryctolagus*	150μM H_2O_2	48	0.0134069	-0.19	**1.0000**
Oryctolagus	0μM H_2O_2	*Oryctolagus*	300μM H_2O_2	48	0.0134069	-0.42	**1.0000**

Supplementary Information

Supplementary Table 22. Two-way ANOVA table and effect test followed by Tukey-HSD post-hoc comparisons of passage 2 (P2) *Acomys*, *Mus, Rattus* & *Oryctolagus* radiation treated fibroblasts cultured for 6hrs to test for differences in percent γH2AX+ cells at 3% O_2. *n* = 3/species

**Source**	**DF**	**SS**	**MS**	***F* Ratio**	***P*-value**
Model	15	2.8277609	0.188517	2445.311	<.0001*
Error	32	0.002467	0.000077		
C. Total	47	2.8302279			

**Source**	**DF**	**SS**	**MS**	***F* Ratio**	***P*-value**
Species	3	0.9987643	0.3329214	4318.415	<.0001*
Dose	3	1.723852	0.5746173	7453.519	<.0001*
Species*Dose	9	0.1051446	0.0116827	151.5399	<.0001*

**Species**	**Dose**	**Species**	**Dose**	**DF**	**SE**	***t-ratio***	***P*-value**
Acomys	0 Gy	*Acomys*	5 Gy	32	0.0071691	-3.59	**0.0652**
Acomys	0 Gy	*Acomys*	15 Gy	32	0.0071691	-46.76	<.0001*
Acomys	0 Gy	*Acomys*	30 Gy	32	0.0071691	-54.41	<.0001*
Mus	0 Gy	*Mus*	5 Gy	32	0.0071691	-36.26	<.0001*
Mus	0 Gy	*Mus*	15 Gy	32	0.0071691	-52.70	<.0001*
Mus	0 Gy	*Mus*	30 Gy	32	0.0071691	-68.32	<.0001*
Rattus	0 Gy	*Rattus*	5 Gy	32	0.0071691	-34.72	<.0001*
Rattus	0 Gy	*Rattus*	15 Gy	32	0.0071691	-65.37	<.0001*
Rattus	0 Gy	*Rattus*	30 Gy	32	0.0071691	-81.78	<.0001*
Oryctolagus	0 Gy	*Oryctolagus*	5 Gy	32	0.0071691	-3.33	**0.1173**
Oryctolagus	0 Gy	*Oryctolagus*	15 Gy	32	0.0071691	-53.78	<.0001*
Oryctolagus	0 Gy	*Oryctolagus*	30 Gy	32	0.0071691	-58.32	<.0001*

Supplementary Table 23. Two-way ANOVA table and effect test followed by Tukey-HSD post-hoc comparisons of passage 2 (P2) *Acomys*, *Mus, Rattus* & *Oryctolagus* radiation treated fibroblasts cultured for 6hrs to test for differences in percent p21+ cells at 3% O_2. n = 3/species

**Source**	**DF**	**SS**	**MS**	***F* Ratio**	***P*-value**
Model	15	2.5593722	0.170625	2277.489	<.0001*
Error	32	0.0023974	0.000075		
C. Total	47	2.5617696			

**Source**	**DF**	**SS**	**MS**	***F* Ratio**	***P*-value**
Species	3	0.8422735	0.2807578	3747.538	<.0001*
Dose	3	1.6559254	0.5519751	7367.729	<.0001*
Species*Dose	9	0.0611734	0.006797	90.7265	<.0001*

**Species**	**Dose**	**Species**	**Dose**	**DF**	**SE**	***t-ratio***	***P*-value**
Acomys	0 Gy	*Acomys*	5 Gy	32	0.0070672	-29.30	<.0001*
Acomys	0 Gy	*Acomys*	15 Gy	32	0.0070672	-44.86	<.0001*
Acomys	0 Gy	*Acomys*	30 Gy	32	0.0070672	-59.66	<.0001*
Mus	0 Gy	*Mus*	5 Gy	32	0.0070672	-44.14	<.0001*
Mus	0 Gy	*Mus*	15 Gy	32	0.0070672	-63.57	<.0001*
Mus	0 Gy	*Mus*	30 Gy	32	0.0070672	-87.03	<.0001*
Rattus	0 Gy	*Rattus*	5 Gy	32	0.0070672	-47.42	<.0001*
Rattus	0 Gy	*Rattus*	15 Gy	32	0.0070672	-62.05	<.0001*
Rattus	0 Gy	*Rattus*	30 Gy	32	0.0070672	-82.42	<.0001*
Oryctolagus	0 Gy	*Oryctolagus*	5 Gy	32	0.0070672	-26.76	<.0001*
Oryctolagus	0 Gy	*Oryctolagus*	15 Gy	32	0.0070672	-39.95	<.0001*
Oryctolagus	0 Gy	*Oryctolagus*	30 Gy	32	0.0070672	-57.04	<.0001*

Supplementary Information

Supplementary Table 24. Two-way ANOVA table and effect test followed by Tukey-HSD post-hoc comparisons of passage 2 (P2) *Acomys*, *Mus, Rattus* & *Oryctolagus* radiation treated fibroblasts cultured for 6hrs to test for differences in percent p53+ cells at 3% O_2. n = 3/species

**Source**	**DF**	**SS**	**MS**	***F* Ratio**	***P*-value**
Model	15	2.656466	0.177098	3034.818	<.0001*
Error	32	0.0018674	0.000058		
C. Total	47	2.6583334			

**Source**	**DF**	**SS**	**MS**	***F* Ratio**	***P*-value**
Species	3	0.9043932	0.3014644	5166.014	<.0001*
Dose	3	1.6722163	0.5574054	9551.922	<.0001*
Species*Dose	9	0.0798565	0.0088729	152.0504	<.0001*

**Species**	**Dose**	**Species**	**Dose**	**DF**	**SE**	***t-ratio***	***P*-value**
Acomys	0 Gy	*Acomys*	5 Gy	32	0.0062373	-30.70	<.0001*
Acomys	0 Gy	*Acomys*	15 Gy	32	0.0062373	-48.11	<.0001*
Acomys	0 Gy	*Acomys*	30 Gy	32	0.0062373	-64.45	<.0001*
Mus	0 Gy	*Mus*	5 Gy	32	0.0062373	-50.80	<.0001*
Mus	0 Gy	*Mus*	15 Gy	32	0.0062373	-72.29	<.0001*
Mus	0 Gy	*Mus*	30 Gy	32	0.0062373	-101.73	<.0001*
Rattus	0 Gy	*Rattus*	5 Gy	32	0.0062373	-54.77	<.0001*
Rattus	0 Gy	*Rattus*	15 Gy	32	0.0062373	-72.41	<.0001*
Rattus	0 Gy	*Rattus*	30 Gy	32	0.0062373	-96.36	<.0001*
Oryctolagus	0 Gy	*Oryctolagus*	5 Gy	32	0.0062373	-30.73	<.0001*
Oryctolagus	0 Gy	*Oryctolagus*	15 Gy	32	0.0062373	-44.47	<.0001*
Oryctolagus	0 Gy	*Oryctolagus*	30 Gy	32	0.0062373	-64.07	<.0001*

Supplementary Information

Supplementary Table 25. Two-way ANOVA table and effect test followed by Tukey-HSD post-hoc comparisons of passage 2 (P2) *Acomys*, *Mus, Rattus* & *Oryctolagus* radiation treated fibroblasts cultured for 6hrs to test for differences in percent p16+ cells at 3% O_2. n = 3/species

**Source**	**DF**	**SS**	**MS**	***F* Ratio**	***P*-value**
Model	15	3.1812729	0.212085	1959.108	<.0001*
Error	32	0.0034642	0.000108		
C. Total	47	3.1847371			

**Source**	**DF**	**SS**	**MS**	***F* Ratio**	***P*-value**
Species	3	2.0864985	0.6954995	6424.591	<.0001*
Dose	3	0.7682805	0.2560935	2365.632	<.0001*
Species*Dose	9	0.3264939	0.0362771	335.1053	<.0001*

**Species**	**Dose**	**Species**	**Dose**	**DF**	**SE**	***t-ratio***	***P*-value**
Acomys	0 Gy	*Acomys*	5 Gy	32	0.0084953	-3.01	**0.2210**
Acomys	0 Gy	*Acomys*	15 Gy	32	0.0084953	-9.38	<.0001*
Acomys	0 Gy	*Acomys*	30 Gy	32	0.0084953	-15.26	<.0001*
Mus	0 Gy	*Mus*	5 Gy	32	0.0084953	-32.24	<.0001*
Mus	0 Gy	*Mus*	15 Gy	32	0.0084953	-49.21	<.0001*
Mus	0 Gy	*Mus*	30 Gy	32	0.0084953	-66.43	<.0001*
Rattus	0 Gy	*Rattus*	5 Gy	32	0.0084953	-36.92	<.0001*
Rattus	0 Gy	*Rattus*	15 Gy	32	0.0084953	-47.68	<.0001*
Rattus	0 Gy	*Rattus*	30 Gy	32	0.0084953	-67.45	<.0001*
Oryctolagus	0 Gy	*Oryctolagus*	5 Gy	32	0.0084953	-3.56	**0.0705**
Oryctolagus	0 Gy	*Oryctolagus*	15 Gy	32	0.0084953	-9.20	<.0001*
Oryctolagus	0 Gy	*Oryctolagus*	30 Gy	32	0.0084953	-13.33	<.0001*

Supplementary Information

Supplementary Table 26. Two-way ANOVA table and effect test followed by Tukey-HSD post-hoc comparisons of passage 2 (P2) *Acomys*, *Mus, Rattus* & *Oryctolagus* radiation treated fibroblasts cultured for 6hrs to test for differences in percent p19+ cells at 3% O_2. n = 3/species

**Source**	**DF**	**SS**	**MS**	***F* Ratio**	***P*-value**
Model	15	3.4880324	0.232535	4699.023	<.0001*
Error	32	0.0015835	0.000049		
C. Total	47	3.489616			

**Source**	**DF**	**SS**	**MS**	***F* Ratio**	***P*-value**
Species	3	2.345844	0.781948	15801.42	<.0001*
Dose	3	0.7415837	0.2471946	4995.25	<.0001*
Species*Dose	9	0.4006048	0.0445116	899.4808	<.0001*

**Species**	**Dose**	**Species**	**Dose**	**DF**	**SE**	***t-ratio***	***P*-value**
Acomys	0 Gy	*Acomys*	5 Gy	32	0.0057437	-3.51	**0.0782**
Acomys	0 Gy	*Acomys*	15 Gy	32	0.0057437	-6.37	<.0001*
Acomys	0 Gy	*Acomys*	30 Gy	32	0.0057437	-18.19	<.0001*
Mus	0 Gy	*Mus*	5 Gy	32	0.0057437	-51.35	<.0001*
Mus	0 Gy	*Mus*	15 Gy	32	0.0057437	-76.71	<.0001*
Mus	0 Gy	*Mus*	30 Gy	32	0.0057437	-99.27	<.0001*
Rattus	0 Gy	*Rattus*	5 Gy	32	0.0057437	-52.68	<.0001*
Rattus	0 Gy	*Rattus*	15 Gy	32	0.0057437	-76.51	<.0001*
Rattus	0 Gy	*Rattus*	30 Gy	32	0.0057437	-102.93	<.0001*
Oryctolagus	0 Gy	*Oryctolagus*	5 Gy	32	0.0057437	-3.68	**0.0529**
Oryctolagus	0 Gy	*Oryctolagus*	15 Gy	32	0.0057437	-7.44	<.0001*
Oryctolagus	0 Gy	*Oryctolagus*	30 Gy	32	0.0057437	-16.17	<.0001*

Supplementary Information

Supplementary Table 27. ANOVA table and effect test followed by t test multiple comparisons of passage 2 (P2) *Acomys*, *Mus, Rattus* & *Oryctolagus* H_2O_2 treated fibroblasts cells to test for differences in basal respiration of mitochondria. *n* = 6/species

**Source**	**DF**	**SS**	**MS**	***F* Ratio**	***P*-value**
Model	7	39412.846	5630.41	15.8779	<.0001*
Error	40	14184.292	354.61		
C. Total	47	53597.138			

**Source**	**DF**	**SS**	**MS**	***F* Ratio**	***P*-value**
Species	3	3	31981.751	30.0631	<.0001*
concentration	1	1	4495.172	12.6765	0.0010*
Species*concentration	3	3	2935.924	2.7598	**0.0546**

**Species**	**Concentration**	**Species**	**Concentration**	**DF**	**SE**	***t-ratio***	***P*-value**
Acomys	0μM H_2O_2	*Acomys*	300μM H_2O_2	40	10.87209	0.41	**0.6876**
Mus	0μM H_2O_2	*Mus*	300μM H_2O_2	40	10.87209	2.36	0.0232*
Rattus	0μM H_2O_2	*Rattus*	300μM H_2O_2	40	10.87209	3.87	.0004*
Oryctolagus	0μM H_2O_2	*Oryctolagus*	300μM H_2O_2	40	10.87209	0.48	**0.6311**

Supplementary Information

Supplementary Table 28. ANOVA table and effect test followed by t test multiple comparisons of passage 2 (P2) *Acomys*, *Mus, Rattus* & *Oryctolagus* H_2O_2 treated fibroblasts cells to test for differences in ATP production of mitochondria. n = 6/species

**Source**	**DF**	**SS**	**MS**	***F* Ratio**	***P*-value**
Model	7	36563.875	5223.41	25.2168	<.0001*
Error	40	8285.617	207.14		
C. Total	47	44849.492			

**Source**	**DF**	**SS**	**MS**	***F* Ratio**	***P*-value**
Species	3	3	23282.918	37.4672	<.0001*
concentration	1	1	6503.979	31.3989	<.0001*
Species*concentration	3	3	6776.978	10.9056	<.0001*

**Species**	**Concentration**	**Species**	**Concentration**	**DF**	**SE**	***t-ratio***	***P*-value**
Acomys	0μM H_2O_2	*Acomys*	300μM H_2O_2	40	8.309441	0.35	**0.7296**
Mus	0μM H_2O_2	*Mus*	300μM H_2O_2	40	8.309441	2.88	0.0064*
Rattus	0μM H_2O_2	*Rattus*	300μM H_2O_2	40	8.309441	7.44	<.0001*
Oryctolagus	0μM H_2O_2	*Oryctolagus*	300μM H_2O_2	40	8.309441	0.54	**0.5955**

Supplementary Table 29. ANOVA table and effect test followed by t test multiple comparisons of passage 2 (P2) *Acomys*, *Mus, Rattus* & *Oryctolagus* H_2O_2 treated fibroblasts cells to test for differences in maximal respiration of mitochondria. *n* = 6/species

**Source**	**DF**	**SS**	**MS**	***F* Ratio**	***P*-value**
Model	7	375941.61	53705.9	20.2582	<.0001*
Error	40	106042.79	2651.1		
C. Total	47	481984.40			

**Source**	**DF**	**SS**	**MS**	***F* Ratio**	***P*-value**
Species	3	3	281276.65	35.3664	<.0001*
concentration	1	1	54210.55	20.4486	<.0001*
Species*concentration	3	3	40454.40	5.0866	0.0045*

**Species**	**Concentration**	**Species**	**Concentration**	**DF**	**SE**	***t-ratio***	***P*-value**
Acomys	0μM H_2O_2	*Acomys*	300μM H_2O_2	40	29.72692	0.09	**0.9288**
Mus	0μM H_2O_2	*Mus*	300μM H_2O_2	40	29.72692	3.22	0.0026*
Rattus	0μM H_2O_2	*Rattus*	300μM H_2O_2	40	29.72692	4.98	<.0001*
Oryctolagus	0μM H_2O_2	*Oryctolagus*	300μM H_2O_2	40	29.72692	0.76	**0.4514**

Supplementary Information

Supplementary Table 30. ANOVA table and effect test followed by t test multiple comparisons of passage 2 (P2) *Acomys*, *Mus, Rattus* & *Oryctolagus* H_2O_2 treated fibroblasts cells to test for differences in spare respiratory capacity of mitochondria. *n* = 6/species

**Source**	**DF**	**SS**	**MS**	***F* Ratio**	***P*-value**
Model	7	176561.91	25223.1	15.0920	<.0001*
Error	40	66851.81	1671.3		
C. Total	47	243413.73			

**Source**	**DF**	**SS**	**MS**	***F* Ratio**	***P*-value**
Species	3	3	127231.96	25.3759	<.0001*
concentration	1	1	27712.43	16.5814	0.0002*
Species*concentration	3	3	21617.52	4.3115	0.0100*

**Species**	**Concentration**	**Species**	**Concentration**	**DF**	**SE**	***t-ratio***	***P*-value**
Acomys	0μM H_2O_2	*Acomys*	300μM H_2O_2	40	23.60293	-0.08	**0.9356**
Mus	0μM H_2O_2	*Mus*	300μM H_2O_2	40	23.60293	2.96	0.0051*
Rattus	0μM H_2O_2	*Rattus*	300μM H_2O_2	40	23.60293	4.49	<.0001*
Oryctolagus	0μM H_2O_2	*Oryctolagus*	300μM H_2O_2	40	23.60293	0.78	**0.4420**

Supplementary Information

Supplementary Table 31. ANOVA table and effect test followed by t test multiple comparisons of isolated mitochondria samples from H_2O_2 treated fibroblasts cells of passage 2 (P2) *Acomys*, *Mus, Rattus* & *Oryctolagus* to test for differences in State III respiration rate of isolated mitochondria. *n* = 3/species

**Source**	**DF**	**SS**	**MS**	***F* Ratio**	***P*-value**
Model	7	220184.06	31454.9	19.1281	<.0001*
Error	16	26310.87	1644.4		
C. Total	23	246494.93			

**Source**	**DF**	**SS**	**MS**	***F* Ratio**	***P*-value**
Species	3	3	197383.62	40.0106	<.0001*
concentration	1	1	11494.22	6.9898	0.0177*
Species*concentration	3	3	11306.22	2.2918	**0.1172**

**Species**	**Concentration**	**Species**	**Concentration**	**DF**	**SE**	***t-ratio***	***P*-value**
Acomys	0μM H_2O_2	*Acomys*	300μM H_2O_2	16	33.11022	0.04	**0.9676**
Mus	0μM H_2O_2	*Mus*	300μM H_2O_2	16	33.11022	2.39	0.0292*
Rattus	0μM H_2O_2	*Rattus*	300μM H_2O_2	16	33.11022	2.85	0.0116*
Oryctolagus	0μM H_2O_2	*Oryctolagus*	300μM H_2O_2	16	33.11022	0.00	**0.9996**

Supplementary Information

Supplementary Table 32. ANOVA table and effect test followed by t test multiple comparisons of isolated mitochondria samples from H_2O_2 treated fibroblasts cells of passage 2 (P2) *Acomys*, *Mus, Rattus* & *Oryctolagus* to test for differences in RCR of isolated mitochondria. *n* = 3/species

**Source**	**DF**	**SS**	**MS**	***F* Ratio**	***P*-value**
Model	7	183.78151	26.2545	19.1558	<.0001*
Error	16	21.92925	1.3706		
C. Total	23	205.71076			

**Source**	**DF**	**SS**	**MS**	***F* Ratio**	***P*-value**
Species	3	3	158.40913	38.5261	<.0001*
concentration	1	1	15.71894	11.4688	0.0038*
Species*concentration	3	3	9.65343	2.3478	**0.1112**

**Species**	**Concentration**	**Species**	**Concentration**	**DF**	**SE**	***t-ratio***	***P*-value**
Acomys	0μM H_2O_2	*Acomys*	300μM H_2O_2	16	0.9558864	0.53	**0.6005**
Mus	0μM H_2O_2	*Mus*	300μM H_2O_2	16	0.9558864	3.41	0.0035*
Rattus	0μM H_2O_2	*Rattus*	300μM H_2O_2	16	0.9558864	2.55	0.0215*
Oryctolagus	0μM H_2O_2	*Oryctolagus*	300μM H_2O_2	16	0.9558864	0.28	**0.7856**

Supplementary Information

Supplementary Table 33. Linear model (from 0.5hrs to 2.5hrs) followed by t test multiple comparisons of H_2O_2 treatment on HyPer transfected cells of passage 2 (P2) *Acomys*, *Mus, Rattus* & *Oryctolagus* to test for differences in fluorescent intensities ratio (F/F0). *n* = 3/species

**Source**	**DF**	***F* Ratio**	***P*-value**
Species	3	8.6390	0.0069*
Passages	1	622.5004	<.0001*
Species*Passages	3	3.9693	0.0137*

**Species**	**-Species**	**DF**	**SE**	***t-ratio***	***P*-value**
Acomys	*Mus*	12	0.068320	3.13	0.0139*
Acomys	*Rattus*	12	0.068320	4.18	0.0031*
Acomys	*Oryctolagus*	12	0.068320	0.28	**0.7840**
Oryctolagus	*Mus*	12	0.068320	-2.85	0.0215*
Oryctolagus	*Rattus*	12	0.068320	3.89	0.0045*
Mus	*Rattus*	12	0.068320	-1.04	**0.3246**

Supplementary Information

Supplementary Table 34. ANOVA table followed by t test multiple comparisons of fluorescent intensities ratio of H_2O_2 treatment on HyPer transfected cells of passage 2 (P2) *Acomys*, *Mus, Rattus* & *Oryctolagus* between 0.5hrs to 4hrs. ANOVA table and t test multiple comparisons to check the significant changes after 2.5hrs, 4hrs and 4.5hrs among all the 4 species. *n* = 3/species

ANOVA table and t-test at 0.5hrs

**Source**	**DF**	**SS**	**MS**	***F* Ratio**	***P*-value**
Species	3	0.05971265	0.019904	1.2153	**0.3651**
Error	8	0.13101999	0.016377		
C. Total	11	0.19073264			

**Species**	**-Species**	**DF**	**SE**	***t-ratio***	***P*-value**
Acomys	*Mus*	12	0.1044908	1.51	**0.1680**
Acomys	*Rattus*	12	0.1044908	1.54	**0.1610**
Acomys	*Oryctolagus*	12	0.1044908	0.42	**0.6786**
Oryctolagus	*Mus*	12	0.1044908	-1.08	**0.3091**
Oryctolagus	*Rattus*	12	0.1044908	1.11	**0.2973**
Mus	*Rattus*	12	0.1044908	0.02	**0.9778**

ANOVA table and t-test at 1 hr

**Source**	**DF**	**SS**	**MS**	***F* Ratio**	***P*-value**
Species	3	0.23363763	0.077879	10.9944	0.0033*
Error	8	0.05666813	0.007084		
C. Total	11	0.29030577			

**Species**	**-Species**	**DF**	**SE**	***t-ratio***	***P*-value**
Acomys	*Mus*	12	0.0687193	3.07	0.0152*
Acomys	*Rattus*	12	0.0687193	4.89	0.0012*
Acomys	*Oryctolagus*	12	0.0687193	0.28	**0.7857**
Oryctolagus	*Mus*	12	0.0687193	-2.79	0.0233*
Oryctolagus	*Rattus*	12	0.0687193	4.61	0.0017*
Mus	*Rattus*	12	0.0687193	1.81	**0.1065**

Supplementary Information

ANOVA table and t-test at 1.5hrs

**Source**	**DF**	**SS**	**MS**	***F*** **Ratio**	***P*****-value**
Species	3	0.17146380	0.057155	7.5375	0.0102*
Error	8	0.06066138	0.007583		
C. Total	11	0.23212518			

**Species**	**-Species**	**DF**	**SE**	***t-ratio***	***P*****-value**
Acomys	*Mus*	12	0.0710993	2.97	0.0177*
Acomys	*Rattus*	12	0.0710993	3.46	0.0085*
Acomys	*Oryctolagus*	12	0.0710993	-0.23	**0.8186**
Oryctolagus	*Mus*	12	0.0710993	-3.21	0.0124*
Oryctolagus	*Rattus*	12	0.0710993	3.70	0.0060*
Mus	*Rattus*	12	0.0710993	0.49	**0.6368**

ANOVA table and t-test at 2hrs

**Source**	**DF**	**SS**	**MS**	***F*** **Ratio**	***P*****-value**
Species	3	0.25438021	0.084793	12.5823	0.0021*
Error	8	0.05391295	0.006739		
C. Total	11	0.30829317			

**Species**	**-Species**	**DF**	**SE**	***t-ratio***	***P*****-value**
Acomys	*Mus*	12	0.0670280	3.79	0.0053*
Acomys	*Rattus*	12	0.0670280	5.12	0.0009*
Acomys	*Oryctolagus*	12	0.0670280	0.45	**0.6584**
Oryctolagus	*Mus*	12	0.0670280	-3.33	0.0103*
Oryctolagus	*Rattus*	12	0.0670280	4.66	0.0016*
Mus	*Rattus*	12	0.0670280	1.33	**0.2198**

Supplementary Information

ANOVA table and t-test at 2.5hrs

Source	DF	SS	MS	*F* Ratio	*P*-value
Species	3	0.25033576	0.083445	12.0277	0.0025*
Error	8	0.05550192	0.006938		
C. Total	11	0.30583768			

Species	-Species	DF	SE	*t-ratio*	*P*-value
Acomys	*Mus*	12	0.0680085	3.45	0.0087*
Acomys	*Rattus*	12	0.0680085	5.01	0.0010*
Acomys	*Oryctolagus*	12	0.0680085	0.27	**0.7908**
Oryctolagus	*Mus*	12	0.0680085	-3.17	0.0131*
Oryctolagus	*Rattus*	12	0.0680085	4.74	0.0015*
Mus	*Rattus*	12	0.0680085	1.56	**0.1563**

ANOVA table and t-test at 3hrs

Source	DF	SS	MS	*F* Ratio	*P*-value
Species	3	0.21603515	0.072012	12.7642	0.0020*
Error	8	0.04513347	0.005642		
C. Total	11	0.26116862			

Species	-Species	DF	SE	*t-ratio*	*P*-value
Acomys	*Mus*	12	0.0613280	3.49	0.0082*
Acomys	*Rattus*	12	0.0613280	5.05	0.0010*
Acomys	*Oryctolagus*	12	0.0613280	0.07	**0.9386**
Oryctolagus	*Mus*	12	0.0613280	-3.41	0.0092*
Oryctolagus	*Rattus*	12	0.0613280	4.97	0.0011*
Mus	*Rattus*	12	0.0613280	1.56	**0.1564**

Supplementary Information

ANOVA table and t-test at 3.5hrs

Source	DF	SS	MS	*F* Ratio	*P*-value
Species	3	0.15282042	0.050940	9.7324	0.0048*
Error	8	0.04187277	0.005234		
C. Total	11	0.19469318			

Species	-Species	DF	SE	*t-ratio*	*P*-value
Acomys	*Mus*	12	0.0590711	3.15	0.0135*
Acomys	*Rattus*	12	0.0590711	4.44	0.0022*
Acomys	*Oryctolagus*	12	0.0590711	0.17	**0.8644**
Oryctolagus	*Mus*	12	0.0590711	2.97	0.0177*
Oryctolagus	*Rattus*	12	0.0590711	4.26	0.0027*
Mus	*Rattus*	12	0.0590711	1.28	**0.2340**

ANOVA table and t-test at 4hrs

Source	DF	SS	MS	*F* Ratio	*P*-value
Species	3	0.11812743	0.039376	14.4922	0.0013*
Error	8	0.02173626	0.002717		
C. Total	11	0.13986369			

Species	-Species	DF	SE	*t-ratio*	*P*-value
Acomys	*Mus*	12	0.0425600	3.90	0.0045*
Acomys	*Rattus*	12	0.0425600	5.61	0.0005*
Acomys	*Oryctolagus*	12	0.0425600	0.54	**0.5989**
Oryctolagus	*Mus*	12	0.0425600	-3.33	0.0100*
Oryctolagus	*Rattus*	12	0.0425600	5.06	0.0010*
Mus	*Rattus*	12	0.0425600	1.71	**0.1247**

Supplementary Information

ANOVA table and t test multiple comparisons table

**Source**	**DF**	**SS**	**MS**	***F* Ratio**	***P*-value**
Species	51	8.4621935	0.165925	24.5918	<.0001
Error	104	0.7017067	0.006747		
C. Total	155	9.1639002			

**Species**	**Time**	**Species**	**Time**	**DF**	**SE**	***t-ratio***	***P*-value**
Acomys	0hr	*Acomys*	2.5hrs	155	0.0670680	-1.42	**0.1592**
Oryctolagus	0hr	*Oryctolagus*	2.5hrs	155	0.0670680	-1.70	**0.0929**
Mus	0hr	*Mus*	4hrs	155	0.0670680	-3.06	0.0028*
Rattus	0hr	*Rattus*	4hrs	155	0.0670680	-3.63	0.0004*
Mus	0hr	*Mus*	5hrs	155	0.0670680	-1.74	**0.0853**
Rattus	0hr	*Rattus*	5hrs	155	0.0670680	-1.91	**0.0588**

Supplementary Information

Supplementary Table 35. Two-way ANOVA table and effect test followed by Tukey-HSD post-hoc comparisons of passage 2 (P2) *Acomys*, *Mus, Rattus* & *Oryctolagus* H_2O_2 treated fibroblasts lysates to test for differences in catalase enzyme activity. We also compared regenerators and non-regenerators groups through LS Means test for changes in catalase activity. *n* = 3/species

**Source**	**DF**	**SS**	**MS**	***F* Ratio**	***P*-value**
Model	7	23.010470	3.28721	97.7947	<.0001*
Error	16	0.537814	0.03361		
C. Total	23	23.548284			

**Source**	**DF**	**SS**	**MS**	***F* Ratio**	***P*-value**
Species	3	3	0.393328	3.9005	0.0288*
concentration	1	1	21.901620	651.5749	<.0001*
Species*concentration	3	3	0.715522	7.0956	0.0030*

**Species**	**Concentration**	**Species**	**Concentration**	**DF**	**SE**	***t-ratio***	***P*-value**
Acomys	0μM H_2O_2	*Mus*	0μM H_2O_2	16	0.1496960	-0.86	**0.9854**
Acomys	0μM H_2O_2	*Rattus*	0μM H_2O_2	16	0.1496960	0.15	**1.0000**
Acomys	0μM H_2O_2	*Oryctolagus*	0μM H_2O_2	16	0.1496960	0.86	**0.9862**
Mus	0μM H_2O_2	*Rattus*	0μM H_2O_2	16	0.1496960	1.01	**0.9657**
Mus	0μM H_2O_2	*Oryctolagus*	0μM H_2O_2	16	0.1496960	1.72	**0.6754**
Oryctolagus	0μM H_2O_2	*Rattus*	0μM H_2O_2	16	0.1496960	-0.71	**0.9955**
Acomys	0μM H_2O_2	*Acomys*	300μM H_2O_2	16	0.1496960	10.23	<.0001*
Mus	0μM H_2O_2	*Mus*	300μM H_2O_2	16	0.1496960	15.58	<.0001*
Rattus	0μM H_2O_2	*Rattus*	300μM H_2O_2	16	0.1496960	14.48	<.0001*
Oryctolagus	0μM H_2O_2	*Oryctolagus*	300μM H_2O_2	16	0.1496960	10.76	<.0001*

**SS**	**NumDF**	**Den DF**	***F* Ratio**	***P*-value**
0.6907673705	1	16	20.5504	0.0003*

Supplementary Information

Supplementary Table 36. Two-way ANOVA table and effect test followed by Tukey-HSD post-hoc comparisons of passage 2 (P2) *Acomys*, *Mus, Rattus* & *Oryctolagus* H_2O_2 treated fibroblasts lysates to test for differences in GPx enzyme activity. We also compared regenerators and non-regenerators groups through LS Means test for changes in GPx activity. *n* = 3/species

**Source**	**DF**	**SS**	**MS**	***F* Ratio**	***P*-value**
Model	7	162.55943	23.2228	172.3622	<.0001*
Error	16	2.15572	0.1347		
C. Total	23	164.71515			

**Source**	**DF**	**SS**	**MS**	***F* Ratio**	***P*-value**
Species	3	3	36.32053	89.8584	<.0001*
concentration	1	1	102.18336	758.4169	<.0001*
Species*concentration	3	3	24.05555	59.5144	<.0001*

**Species**	**Concentration**	**Species**	**Concentration**	**DF**	**SE**	***t-ratio***	***P*-value**
Acomys	0μM H_2O_2	*Mus*	0μM H_2O_2	16	0.2997026	1.47	**0.8128**
Acomys	0μM H_2O_2	*Rattus*	0μM H_2O_2	16	0.2997026	1.56	**0.7634**
Acomys	0μM H_2O_2	*Oryctolagus*	0μM H_2O_2	16	0.2997026	-0.08	**1.0000**
Mus	0μM H_2O_2	*Rattus*	0μM H_2O_2	16	0.2997026	0.10	**1.0000**
Mus	0μM H_2O_2	*Oryctolagus*	0μM H_2O_2	16	0.2997026	-1.54	**0.7744**
Oryctolagus	0μM H_2O_2	*Rattus*	0μM H_2O_2	16	0.2997026	1.64	**0.7218**
Acomys	0μM H_2O_2	*Acomys*	300μM H_2O_2	16	0.2997026	-22.00	<.0001*
Mus	0μM H_2O_2	*Mus*	300μM H_2O_2	16	0.2997026	-7.68	<.0001*
Rattus	0μM H_2O_2	*Rattus*	300μM H_2O_2	16	0.2997026	-6.72	.0001*
Oryctolagus	0μM H_2O_2	*Oryctolagus*	300μM H_2O_2	16	0.2997026	-18.68	<.0001*

**SS**	**NumDF**	**Den DF**	***F* Ratio**	***P*-value**
23.25	1	16	172.5647	<.0001*

Supplementary Information

Supplementary Table 37. ANOVA table and t-test of 1hr pretreated NAC followed by 2hrs H_2O_2 treated fibroblasts cells of passage 2 (P2) from *Mus* and *Rattus* to test for differences in basal respiration. *n* = 5/species

**Source**	**DF**	**SS**	**MS**	***F* Ratio**	***P*-value**
Model	2	1073.1596	536.580	5.9922	0.0157*
Error	12	1074.5647	89.547		
C. Total	14	2147.7242			

**Species**	**Levels**	**-Levels**	**Difference**	**SE**	***P*-value**
Mus	0uM H_2O_2	300uM H_2O_2	20.67310	5.984883	0.0048*
Mus	0uM H_2O_2	2mM NAC +300uM H_2O_2	11.52614	5.984883	**0.0781**
Mus	2mM NAC +300uM H_2O_2	300uM H_2O_2	9.14696	5.984883	**0.1523**

**Source**	**DF**	**SS**	**MS**	***F* Ratio**	***P*-value**
Model	2	3316.722	1658.36	2.7081	**0.1070**
Error	12	7348.536	612.38		
C. Total	14	10665.258			

**Species**	**Levels**	**-Levels**	**Difference**	**SE**	***P*-value**
Rattus	0uM H_2O_2	300uM H_2O_2	35.41301	15.65092	0.0430*
Rattus	0uM H_2O_2	2mM NAC +300uM H_2O_2	25.08594	15.65092	**0.1350**
Rattus	2mM NAC +300uM H_2O_2	300uM H_2O_2	10.32707	15.65092	**0.5218**

Supplementary Information

Supplementary Table 38. ANOVA table and t-test of 1hr pretreated NAC followed by 2hrs H_2O_2 treated fibroblasts cells of passage 2 (P2) from *Mus* and *Rattus* to test for differences in Maximal respiration. *n* = 5/species

**Source**	**DF**	**SS**	**MS**	***F* Ratio**	***P*-value**
Model	2	37354.409	18677.2	8.5244	0.0050*
Error	12	26292.324	2191.0		
C. Total	14	63646.734			

**Species**	**Levels**	**-Levels**	**Difference**	**SE**	***P*-value**
Mus	0uM H_2O_2	300uM H_2O_2	121.8965	29.60424	0.0014*
Mus	0uM H_2O_2	2mM NAC +300uM H_2O_2	68.8380	29.60424	0.0384*
Mus	2mM NAC +300uM H_2O_2	300uM H_2O_2	53.0585	29.60424	**0.0983**

**Source**	**DF**	**SS**	**MS**	***F* Ratio**	***P*-value**
Model	2	44973.912	22487.0	7.6320	0.0073*
Error	12	35356.757	2946.4		
C. Total	14	80330.669			

**Species**	**Levels**	**-Levels**	**Difference**	**SE**	***P*-value**
Rattus	0uM H_2O_2	300uM H_2O_2	124.4036	34.33014	0.0035*
Rattus	0uM H_2O_2	2mM NAC +300uM H_2O_2	18.7853	34.33014	**0.5943**
Rattus	2mM NAC +300uM H_2O_2	300uM H_2O_2	105.6182	34.33014	0.0096*

Supplementary Information

Supplementary Table 39. ANOVA table and t-test of pretreated NAC followed by H_2O_2 treated fibroblasts cells of passage 2 (P2) from *Mus* and *Rattus* to test for differences in ATP production respiration. n = 5/species

**Source**	**DF**	**SS**	**MS**	***F* Ratio**	***P*-value**
Model	2	1507.5296	753.765	10.5917	0.0022*
Error	12	853.9838	71.165		
C. Total	14	2361.5135			

**Species**	**Levels**	**-Levels**	**Difference**	**SE**	***P*-value**
Mus	0uM H_2O_2	300uM H_2O_2	24.39231	5.335366	0.0006*
Mus	0uM H_2O_2	2mM NAC +300uM H_2O_2	9.74255	5.335366	**0.0928**
Mus	2mM NAC +300uM H_2O_2	300uM H_2O_2	14.64976	5.335366	0.0177*

**Source**	**DF**	**SS**	**MS**	***F* Ratio**	***P*-value**
Model	2	13983.723	6991.86	11.1963	0.0018*
Error	12	7493.732	624.48		
C. Total	14	21477.455			

**Species**	**Levels**	**-Levels**	**Difference**	**SE**	***P*-value**
Rattus	0uM H_2O_2	300uM H_2O_2	74.28443	15.80478	0.0005*
Rattus	0uM H_2O_2	2mM NAC +300uM H_2O_2	29.62661	15.80478	**0.0854**
Rattus	2mM NAC +300uM H_2O_2	300uM H_2O_2	44.65782	15.80478	0.0153*

Supplementary Information

Supplementary Table 40. ANOVA table and t-test of 1hr pretreated NAC followed by 2hrs H_2O_2 treated fibroblasts cells of passage 2 (P2) from *Mus* and *Rattus* to test for differences in Spare respiratory capacity. *n* = 5/species

**Source**	**DF**	**SS**	**MS**	***F* Ratio**	***P*-value**
Model	2	17600.694	8800.35	4.2258	0.0408*
Error	12	24990.343	2082.53		
C. Total	14	42591.037			

**Species**	**Levels**	**-Levels**	**Difference**	**SE**	***P*-value**
Mus	0uM H_2O_2	300uM H_2O_2	83.24828	28.86194	0.0137*
Mus	0uM H_2O_2	2mM NAC +300uM H_2O_2	32.54110	28.86194	**0.2816**
Mus	2mM NAC +300uM H_2O_2	300uM H_2O_2	50.70717	28.86194	**0.1044**

**Source**	**DF**	**SS**	**MS**	***F* Ratio**	***P*-value**
Model	2	50636.361	25318.2	14.4430	0.0006*
Error	12	21035.673	1753.0		
C. Total	14	71672.034			

**Species**	**Levels**	**-Levels**	**Difference**	**SE**	***P*-value**
Rattus	0uM H_2O_2	300uM H_2O_2	137.4766	26.47998	0.0002*
Rattus	0uM H_2O_2	2mM NAC +300uM H_2O_2	36.8629	26.47998	**0.1892**
Rattus	2mM NAC +300uM H_2O_2	300uM H_2O_2	100.6137	26.47998	0.0025*

Supplementary Information

Supplementary Table 41. ANOVA table and t-test of 1hr pretreated NAC followed by 2hrs H_2O_2 treated fibroblasts cells of passage 2 (P2) from *Mus* and *Rattus* to test for differences in %p21+ cells. *n* = 5/species

**Source**	**DF**	**SS**	**MS**	***F* Ratio**	***P*-value**
Model	2	6801.2793	3400.64	1309.3470	<.0001*
Error	9	23.3748	2.60		
C. Total	11	6824.6541			

**Species**	**Levels**	**-Levels**	**Difference**	**SE**	***P*-value**
Mus	0uM H_2O_2	300uM H_2O_2	52.66000	1.139562	<.0001*
Mus	0uM H_2O_2	2mM NAC +300uM H_2O_2	4.63500	1.139562	0.0071*
Mus	2mM NAC +300uM H_2O_2	300uM H_2O_2	48.02500	1.139562	<.0001*

**Source**	**DF**	**SS**	**MS**	***F* Ratio**	***P*-value**
Model	2	4275.3534	2137.68	258.1558	<.0001*
Error	9	74.5251	8.28		
C. Total	11	4349.8785			

**Species**	**Levels**	**-Levels**	**Difference**	**SE**	***P*-value**
Rattus	0uM H_2O_2	300uM H_2O_2	42.25500	2.034769	<.0001*
Rattus	0uM H_2O_2	2mM NAC +300uM H_2O_2	4.87500	2.034769	**0.0924**
Rattus	2mM NAC +300uM H_2O_2	300uM H_2O_2	37.38000	2.034769	<.0001*

Supplementary Information

Supplementary Table 42. ANOVA table and t-test of 1hr pretreated NAC followed by 2hrs by H_2O_2 treated fibroblasts cells of passage 2 (P2) from *Mus* and *Rattus* to test for differences in % SAβ-gal+ cells. *n* = 5/species

**Source**	**DF**	**SS**	**MS**	***F* Ratio**	***P*-value**
Model	2	2351.2193	1175.61	669.5451	<.0001*
Error	9	15.8025	1.76		
C. Total	11	2367.0218			

**Species**	**Levels**	**-Levels**	**Difference**	**SE**	***P*-value**
Mus	0uM H_2O_2	300uM H_2O_2	30.62500	0.9369721	<.0001*
Mus	0uM H_2O_2	2mM NAC +300uM H_2O_2	1.96000	0.9369721	**0.1465**
Mus	2mM NAC +300uM H_2O_2	300uM H_2O_2	28.66500	0.9369721	<.0001*

**Source**	**DF**	**SS**	**MS**	***F* Ratio**	***P*-value**
Model	2	767.09947	383.550	125.3335	<.0001*
Error	9	27.54210	3.060		
C. Total	11	794.64157			

**Species**	**Levels**	**-Levels**	**Difference**	**SE**	***P*-value**
Rattus	0uM H_2O_2	300uM H_2O_2	18.41000	1.236979	<.0001*
Rattus	0uM H_2O_2	2mM NAC +300uM H_2O_2	3.42000	1.236979	**0.0522**
Rattus	2mM NAC +300uM H_2O_2	300uM H_2O_2	14.99000	1.236979	<.0001*
